# Biochemical and functional characterization of *Helicobacter pylori* vesicles

**DOI:** 10.1111/j.1365-2958.2010.07307.x

**Published:** 2010-08-05

**Authors:** Annelie Olofsson, Anna Vallström, Katja Petzold, Nicole Tegtmeyer, Jürgen Schleucher, Sven Carlsson, Rainer Haas, Steffen Backert, Sun Nyunt Wai, Gerhard Gröbner, Anna Arnqvist

**Affiliations:** 1 Department of Medical Biochemistry and Biophysics, Umeå UniversitySE-901 87 Umeå, Sweden; 2 Department of Odontology/Oral Microbiology, Umeå UniversitySE-901 87 Umeå, Sweden; 3 School of Biomolecular and Biomedical Sciences, University College DublinIreland; 4 Max-von-Pettenkofer-Institute of Hygiene and Medical Microbiology, Department of BacteriologyMunich, Germany; 5 Department of Molecular Biology, Umeå UniversitySE-901 87 Umeå, Sweden; 6 Department of Chemistry, Umeå UniversitySE-901 87 Umeå, Sweden

## Abstract

*Helicobacter pylori* can cause peptic ulcer disease and/or gastric cancer. Adhesion of bacteria to the stomach mucosa is an important contributor to the vigour of infection and resulting virulence. *H. pylori* adheres primarily via binding of BabA adhesins to ABO/Lewis b (Leb) blood group antigens and the binding of SabA adhesins to sialyl-Lewis x/a (sLex/a) antigens. Similar to most Gram-negative bacteria, *H. pylori* continuously buds off vesicles and vesicles derived from pathogenic bacteria often include virulence-associated factors. Here we biochemically characterized highly purified *H. pylori* vesicles. Major protein and phospholipid components associated with the vesicles were identified with mass spectroscopy and nuclear magnetic resonance. A subset of virulence factors present was confirmed by immunoblots. Additional functional and biochemical analysis focused on the vesicle BabA and SabA adhesins and their respective interactions to human gastric epithelium. Vesicles exhibit heterogeneity in their protein composition, which were specifically studied in respect to the BabA adhesin. We also demonstrate that the oncoprotein, CagA, is associated with the surface of *H. pylori* vesicles. Thus, we have explored mechanisms for intimate *H. pylori* vesicle–host interactions and found that the vesicles carry effector-promoting properties that are important to disease development.

## Introduction

*Helicobacter pylori* is estimated to have infected more than half of all people worldwide. Of the infected individuals, ∼10% develop peptic ulcer disease and 1–2% develop gastric cancer (Cover *et al*., 2001). Furthermore, individuals infected by *H. pylori* persist as non-symptomatic carriers for their lifetime. Host inflammatory responses and effector molecules produced by *H. pylori-*infected cells cause changes in the gastric mucosa during infection, requiring *H. pylori* to be able to adapt to an ever-changing environment. Several factors with vital roles in the colonization of *H. pylori* and the development of disease have been described. These factors include the urease enzyme, flagella, the vacuolating cytotoxin VacA and the *cag*-pathogenicity island (*cag*PAI), which encodes the CagA protein and a type IV secretion (T4S) machinery that translocates CagA into host cells (Segal *et al*., 1999; Odenbreit *et al*., 2000). Both phosphorylated and non-phosphorylated CagA have been shown to interfere with host signalling pathways and cellular functions (Higashi *et al*., 2002; Hatakeyama, 2006; Suzuki *et al*., 2009).

Adherence is important for persistent infection of a host and is mediated by the binding of bacteria to glycoproteins and/or glycolipids present on the host cell surface. Analysis of gastric biopsy material has shown that *H. pylori* populations are found deep in the mucous layer, and a subset is attached to the surface of epithelial cells (Hessey *et al*., 1990). Recent reports have also suggested *H. pylori* to be localized intracellularly (Semino-Mora *et al*., 2003; Oh *et al*., 2005; Aspholm *et al*., 2006a). Several receptor structures for adherence of *H. pylori* to human gastric epithelial cells have been described, suggesting that *H. pylori* exhibits a multitude of adherence modes, which may change as infection progresses (Gerhard *et al*., 2001). Two functional receptors for *H. pylori*, fucosylated ABO/Lewis b antigens (Leb) (Borén *et al*., 1993; Aspholm-Hurtig *et al*., 2004) and sialyl-Lewis x/a antigens (sLex/a) (Mahdavi *et al*., 2002), have been described in detail. In healthy human gastric epithelium, only minute levels of sialylated glycans are detected, but upon infection by *H. pylori*, increased expression of the inflammation/selectin-associated sLex occurs (Mahdavi *et al*., 2002; Kobayashi *et al*., 2004). Bacterial adherence to host cells is mediated by adhesins on the bacterial cell surface. Correspondingly, the *H. pylori* blood group antigen binding adhesin (BabA) mediates binding to the ABO/Leb receptor structures (Ilver *et al*., 1998), and binding to sLex/a is mediated by the sialic acid binding adhesin (SabA) (Mahdavi *et al*., 2002; Aspholm *et al*., 2006a).

Gram-negative bacteria continuously shed vesicles from the cell surface during their growth. These vesicles are 20–300 nm in size and are surrounded by an outer membrane (OM) layer, which is described to have a composition derived from the bacteria's OM. Upon shedding from the bacterial cell surface, some of the underlying periplasmic and cytoplasmic proteins, as well as DNA and RNA, are contained within the vesicles (Beveridge, 1999; Kuehn and Kesty, 2005; Mashburn-Warren and Whiteley, 2006). The biological role of these vesicles has not been fully elucidated, but is described to be involved in toxin delivery, protein and DNA transfer, and signalling between bacteria. Biochemical characterization of vesicles isolated from pathogenic species has identified virulence-associated factors such as toxins, invasins and host–effector molecules (Horstman and Kuehn, 2000; Wai *et al*., 2003; Kesty and Kuehn, 2004; Post *et al*., 2005; Balsalobre *et al*., 2006; Tan *et al*., 2007; Vidakovics *et al*., 2010). Electron micrographs of plasma from a person infected with *Neisseria meningitidis* serogroup B that died of septic shock showed that numerous vesicles were present (Namork and Brandtzaeg, 2002). *H. pylori* vesicles were found in gastric mucosal biopsy material and shown to contain the VacA cytotoxin (Fiocca *et al*., 1999; Keenan *et al*., 2000; Ricci *et al*., 2005). Recently, peptidoglycan present in *H. pylori* vesicles was described to upregulate NF-κB and Nod1-dependent responses (Kaparakis *et al*., 2010). Although interactions between host cells and bacterial vesicles have been described *in vitro* (Beveridge, 1999; Kuehn and Kesty, 2005), details in the adherence mechanisms are still poorly defined.

Here we provide a detailed biochemical and functional characterization of the vesicles that are released from *H. pylori*. We established protocols for the isolation and purification of *H. pylori* vesicles according to size or density. Major protein and phosholipid components present were identified with nano-liquid chromatography Fourier transform-ion cyclotron resonance mass spectrometry and tandem mass spectrometry analysis (nanoflow LC FT-ICR MS/MS) and high-resolution liquid state ^31^P,^1^H nuclear magnetic resonance (NMR) respectively. We demonstrate that the protein content of *H. pylori* vesicles differs significantly from whole bacterial cells as well as of the OM.

Using immunoblots we confirmed the presence of a series of virulence factors in the vesicles such as adherence components and the oncoprotein CagA. In particular, we focused on biochemical characterization and functional analysis of adherence components present on the surface of *H. pylori* vesicles. We used receptor conjugates for electron microscopy studies and an *in vitro* assay to characterize adherence to human gastric tissue sections. The results of these studies provide valuable insight into the mechanisms involved in bacterial–host interactions as they relate to *H. pylori* vesicles and their intimate interaction with human gastric mucosa.

## Results

### Isolation of *H. pylori* vesicles

**Fig. 1:**
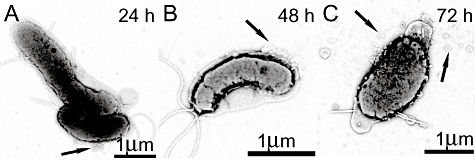
Vesicle production relative to bacterial growth. Electron micrographs of the *H. pylori* strain, CCUG17875, harvested after (A) 24 h; (B) 48 h; and (C) 72 h. Bar length represents 1 µm. Arrow points out vesicles.

To biochemically analyse *H. pylori* vesicles, optimal growth phase conditions for the isolation of vesicles needed to be established. For this purpose, *H. pylori* strain CCUG17875 was harvested at different time points from Brucella blood agar and visualized by electron microscopy to follow changes in morphology and vesicle production (Fig. 1). *H. pylori* collected during the logarithmic growth phase were spiral-shaped and produced small numbers of vesicles (Fig. 1A). Upon entry into stationary phase, *H. pylori* became curved and later doughnut-shaped while producing a greater number of vesicles (Fig. 1B). During late stationary phase, *H. pylori* had become coccoid and produced large amounts of vesicles (Fig. 1C). For further experiments, *H. pylori* were harvested in the early stationary phase since cells were viable, contained low levels of lysed cell debris, and produced sufficient quantities of vesicle material.

**Fig. 2:**
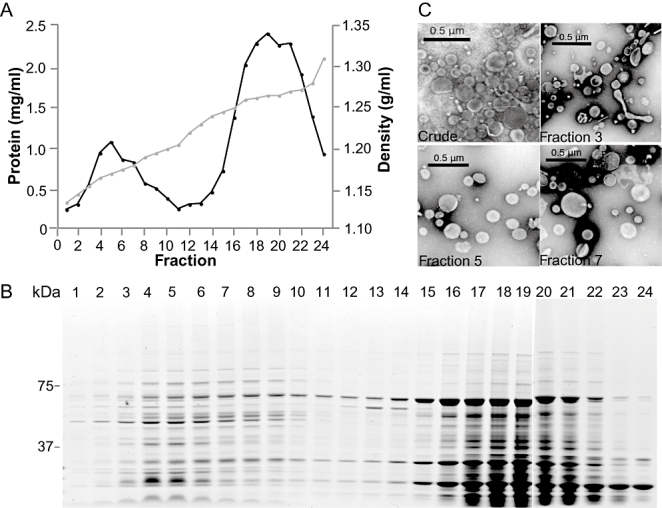
Isolation of *H. pylori* vesicles according to density. Crude *H. pylori* vesicle preparations were subjected to flotation and separated using density gradients. A. The density and protein concentration of each fraction was measured. The densities are depicted in grey and protein concentrations in black. B. The same volume from each fraction was analysed by SDS-PAGE. Fraction 1 corresponds to the top fraction and has the lowest density (1.135 g ml^−1^). C. Electron micrographs of *H. pylori* vesicles from strain CCUG17875, are shown including: crude fraction, fraction 3, 5 and 7. Bar length represents 0.5 µm.

*Helicobacter pylori* vesicles were isolated and purified from crude vesicle extracts by centrifugation to equilibrium in a continuous density gradient (20–60%). Fractions were collected from top to bottom and the gradient formation and protein concentration of each fraction was determined (Fig. 2A). Peaks in protein concentration were identified in fractions 3–9 and fractions 15–24. Equal volumes from each fraction were analysed for protein composition by SDS-PAGE (Fig. 2B). The protein pattern and content varied between fractions. All fractions were also analysed by electron microscopy to identify vesicle-containing fractions (data not shown). Spherical vesicles ranging in size from 20–300 nm dominated in fractions 3–9, and electron micrographs confirmed that these fractions contained purified vesicles free from bacterial debris and soluble proteins compared to crude vesicle preparations (Fig. 2B and C).

**Fig. 3:**
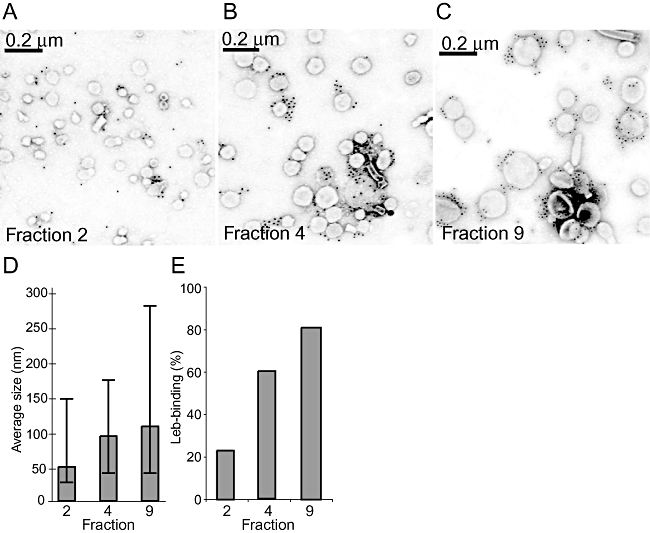
Analysis of *H. pylori* vesicles isolated according to size. Crude *H. pylori* vesicle preparations were separated on a gradient according to size. A–C. Electron micrographs of fractions 2, 4 and 9 that represent the differences in vesicle size achieved by separation. Leb-binding adhesins on the surface of the vesicles are visualized with immunogold labelling of Leb–receptor conjugates. Bar length represents 0.2 µm. D. A bar graph representing the average size of vesicles isolated in fractions 2: 50 nm (*n* = 207); 4: 94 nm (*n* = 146); and 9: 108 nm (*n* = 188). Maximum and minimum ranges for each fraction are represented by the two ends of the vertical lines associated with each bar. E. A bar graph representing the percentage of Leb-binding vesicles present in each fraction, i.e. fraction 2: 23% (*n* = 524); 4: 60% (*n* = 351); and 9: 81% (*n* = 183). The presented data represent one of three independent sets of experiments.

With shorter centrifugation times, vesicles could be separated according to size. A crude vesicle extract was subjected to centrifugation on a continuous 5–15% gradient. Size-separated fractions were then analysed by SDS-PAGE (Fig. S1) and electron microscopy to confirm the separation of vesicles according to size (Fig. 3A–C). The average vesicle size in each fraction was determined. Fraction 2 contained vesicles of 50 nm, fraction 4 vesicles of 94 nm, and fraction 9 vesicles of 108 nm (Fig. 3D).

### Characterization of major phospholipid vesicle components

**Fig. 4:**
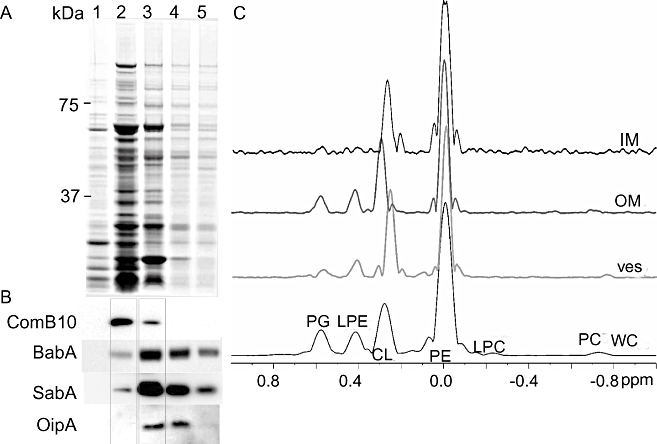
Identification of major phospholipid components in *H. pylori* vesicles. *H. pylori* membranes were fractionated using sucrose density gradient centrifugation without detergents. A. Equal volumes from the top five fractions were analysed by SDS-PAGE. B. Fractions were probed in immunoblots for the IM protein ComB10, and the OM proteins BabA, SabA and OipA respectively. C. Major phospholipids present in whole *H. pylori* bacterial cells (WC) versus inner membrane (IM, fraction 2), outer membrane (OM, fraction 3) and *H. pylori* vesicles (ves) were identified using high-resolution NMR. The displayed ^31^P projections of two-dimensional ^31^P, ^1^H correlation spectra are scaled for equal intensity of the PE signals. Slight shifts in ^31^P positions are due to variations in solvent composition. The following phospholipids were identified: Phosphatidylglycerol (PG), phosphatidyl ethanolamine (PE), lyso PE (LPE), phosphatidylcholine (PC), lyso PC (LPC) and cardiolipin (CL).

Phospholipids present in *H. pylori* vesicles were identified using two-dimensional (2D) ^31^P,^1^H NMR correlation spectra. The type and abundance of phospholipid components present in isolated vesicles, outer and inner membrane (IM) fractions, and whole *H. pylori* cells was determined. By definition, IMs have fewer proteins than OMs, thereby allowing IMs to be separated from OMs using density gradient centrifugation. To localize fractions in which IM and OM dominated, fractions were separated with SDS-PAGE and probed with markers for IM and OM respectively (Fig. 4A and B). Antiserum against ComB10 was used as IM protein marker (Kutter *et al*., 2008) and a strong signal was obtained in fraction 2. Weak or no signals were obtained in fraction 1, 3, 4 and 5. BabA, SabA and OipA are all OM proteins (Ilver *et al*., 1998; Yamaoka *et al*., 2000; Mahdavi *et al*., 2002) and signals were obtained in fractions 3, 4 and 5 (no OipA signal in 5). Fraction 2 was thereby defined as the fraction where IM dominated and fraction 3 defined as the fraction where OM dominated.

Lipids were extracted from whole bacteria, OM (Fig. 4A and B, fraction 3), IM (Fig. 4A and B, fraction 2) and purified vesicles. For analysis of the 2D spectra (Fig. S2), 1D projections were integrated (Fig. 4C). Phosphatidylethanolamine (PE) and cardiolipin (CL) were identified as the major phospholipids present in all fractions. IM fractions contained considerably lower amounts of phosphatidylglycerol (PG) and lyso-phophatidyletanolamine (LPE) compared with the OM or vesicle fractions. Phophatidylcholine (PC) was below the detection limit in IM, but was readily detected in all other fractions (Fig. S2). These data represent the differences in phospholipid composition between the analysed fractions, and suggests that the phospholipid composition of vesicles most closely resembles that of the OM.

*Helicobacter pylori* membranes have been reported to contain cholesterol (Inamoto *et al*., 1993), therefore we used ^13^C,^1^H NMR correlation spectra to quantify the levels of cholesterol present in whole cell and vesicle samples. Relating the phospholipid signals with cholesterol signals resulted in a molar fraction of cholesterol estimated to be 10% relative to that of total phospholipids present in both whole bacterial cells as well as in vesicles (data not shown).

### Characterization of major proteins present in vesicles

**Fig. 5:**
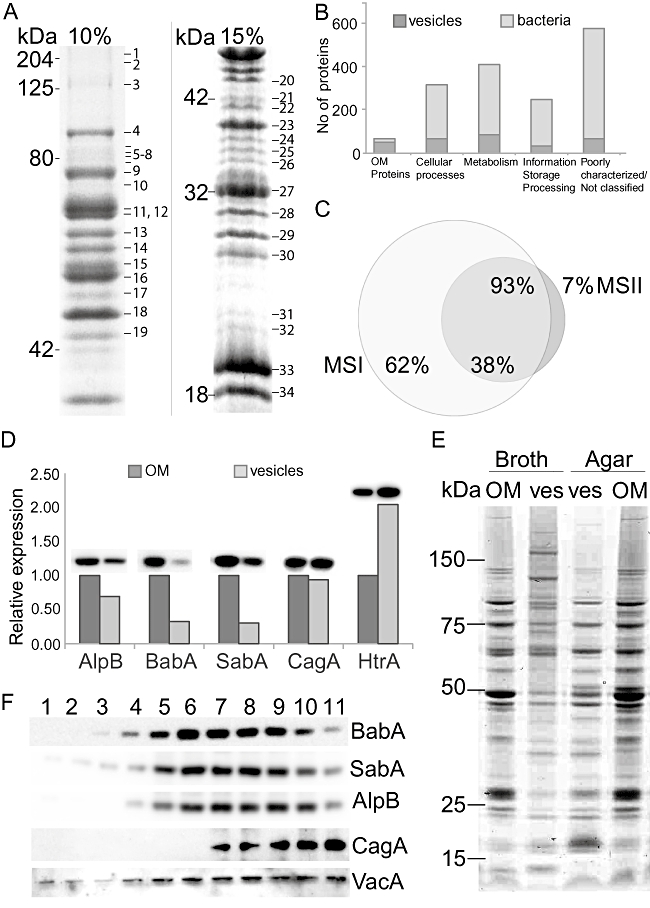
Identification of major protein components in *H. pylori* vesicles. A. Protein extracts of *H. pylori* CCUG17875 vesicles (fractions 3–9) were separated by both 10% and 15% SDS-PAGE to resolve the protein bands present. Thirty-four of the most predominant bands (labelled to the right of each gel lane) were excised and subjected to nanoflow LC FT-ICR MS/MS. Molecular weights (kDa) are marked to the left of each gel lane. B. The identified vesicle proteins were sorted into functional COG classes and outer membrane proteins respectively. Dark grey bar represents vesicle proteins in each class and light grey bars represent number of proteins of each class of the total *H. pylori* proteome. Out of the total *H. pylori* proteome, 77% of all OM proteins were found in the vesicles; 21% of cellular processes; 21% of metabolism; 14% of information, storage and processing; and 12% of poorly classified/not classified. C. Comparison of MS I and MS II analyses presented as a Venn diagram. The two data sets are represented proportional to the size of the data sets. Approximately 38% of the proteins identified in the MS I analysis were found in the MS II analysis, whereas 93% of proteins from the MS II analysis were found in the MS I analysis. D. OM proteins and vesicles were analysed by immunoblot using antibodies specific for the lipoprotein AlpB, the BabA adhesin, the SabA adhesin, the CagA and the HtrA proteins respectively. Signal densities were measured. Signals corresponding to OM were set to 1.0 to correlate the relative expression of signals obtained in vesicles. The ratio of OM versus vesicles were: AlpB: 1:0.7; BabA: 1:0.3; SabA: 1:0.3; CagA: 1:0.9; HtrA: 1:2.0. The immunoblots shown in the figure represent one for each protein of a series of blots. E. *H. pylori* CCUG17875 were grown on Brucella blood agar and in Brucella broth. Protein extracts of OM and of vesicles obtained from the two different growth conditions were analysed for their protein expression patterns by SDS-PAGE. Molecular weights (kDa) are labelled on the far left. F. The same volume from fractions 1–11 of vesicles separated by density were analysed by immunoblotting using antibodies specific for BabA adhesin, SabA adhesin, lipoprotein AlpB, CagA and VacA respectively.

The major protein components of *H. pylori* vesicles were analysed using mass spectrometry (MS). Since many of the *H. pylori* OM proteins are of similar molecular masses, exhibit high pI values and do not resolve on 2D gels, 1D SDS-PAGE was combined with analysis by the sensitive nanoflow LC FT-ICR MS/MS method. Using *H. pylori* vesicles purified according to density, samples with a density of 1.15–1.20 g ml^−1^ from fractions 3–9 were pooled. Initial separation of the samples by SDS-PAGE identified a series of 34 bands that were excised and subjected to peptide tandem mass determination (Fig. 5A). All mass data obtained were searched by MASCOT against all species in the NCBI database. An *H. pylori* proteome consists of approximately 1500 putative proteins (Alm *et al*., 1999; Oh *et al*., 2006; Baltrus *et al*., 2009), and in total 306 different *H. pylori* proteins were identified in the 34 excised bands (Table S1). Identified vesicle proteins were classified according to Clusters of Orthologous Groups (COG) for the total *H. pylori* proteome (Table S1, Fig. 5B). Several *H. pylori* OM proteins have been associated with gastric disease (Yamaoka and Alm, 2008), and therefore *H. pylori* OM proteins are listed as a separate group as classified according to Alm *et al*. (2000). A majority of all OM proteins (77%) were identified in the vesicles (Fig. 5B). We used a sensitive MS method, which may explain why we also found way more cytoplasmic and periplasmic components than previously shown for bacterial vesicles. Several of the identified vesicle proteins are associated with important roles in *H. pylori* colonization and virulence, such as urease subunits, the cytotoxin VacA, CagA, and the BabA and SabA adhesins. We also found γ-glutamyl transpeptidase that similar to the VacA protein has been shown to exhibit immunosuppressive effects (Schmees *et al*., 2007) and the protease HtrA (Löwer *et al*., 2008). Among the series of cytoplasmic proteins we found GroEL, catalase, metabolic and ribosomal proteins.

A second MS analysis (MS II) was performed on an additional vesicle sample of a narrower density interval (1.17–1.18 g ml^−1^). Separation by SDS-PAGE resolved a series of 32 bands that were isolated (Fig. S3) and subjected to nanoflow LC FT-ICR MS/MS analysis. From this sample, 126 different *H. pylori* proteins were identified. Among these, 93% were identical to the previous analysis (Fig. 5C, Table S1) and 7% were specific for MS II (Table S2).

The content of bacterial vesicles have been described to resemble the OM (Beveridge, 1999; Kuehn and Kesty, 2005; Mashburn-Warren and Whiteley, 2006). Since the MS analysis is not quantitative, we wanted to determine the relative amount in a series of virulence-associated proteins in the OM and vesicles. The same amount of protein was analysed in immunoblots for the BabA, SabA and AlpB proteins, in addition to the CagA and the HtrA proteins (Fig. 5D). The expression level of the respective proteins in vesicles was related to the levels in OM (Fig. 5D). The BabA and the SabA adhesins were markedly enriched in the OM relative to the levels in the vesicles. The same enrichment in the OM was not as obvious for the AlpB and the CagA proteins. Interestingly, the relative HtrA level was higher in the vesicles (Fig. 5D).

In contrast to strain CCUG17875, strain P12 has a functional T4S machinery. We probed for a series of T4S associated proteins (CagT, CagX, CagY, CagN, CagM and VirD4) and their presence in vesicles isolated from strains P12 and CCUG17875 (Fig. S4). CagT, CagX, CagN and CagM were all present in vesicles from strain CCUG17875. We also noted a higher amount of these proteins in vesicles from CCUG17875 than P12 vesicles. VirD4 was present in P12 bacterial cells but no protein was detected in P12 vesicles. Additionally, we probed for the OipA protein and showed its presence in both whole cells and vesicles from strain CCUG17875 and P12 (Fig. S4).

Taken together, the results from the MS analysis and the immunoblots support the view that vesicles are comprised of a protein composition that is distinct from whole cells and OM respectively.

Since growth conditions affect protein expression, *H. pylori* vesicles and OM were isolated after growth in Brucella broth cultures and blood agar. The samples were analysed by SDS-PAGE (Fig. 5E) and clearly demonstrate that differences in environmental conditions affect the content of vesicles since the protein pattern of broth and agar differed. In general, the vesicle protein profile resembled that of the OM, but some clear distinctions in the protein pattern were also observed (Fig. 5E).

### Identification of virulence-associated proteins

To identify potential virulence-associated factors present in isolated fractions, immunoblot analysis was performed. Protein extracts from *H. pylori* vesicles separated according to density (i.e. fractions 1–11) were analysed by immunoblot with antibodies specific for the BabA and SabA adhesins, VacA, AlpB and CagA (Fig. 5F). Immunoblot analysis with antibodies specific for the OM protein OipA showed that fractions 5–7 were associated with the OipA protein (data not shown). The results of the immunoblots also verify that vesicles are heterogeneous in their protein content.

### Specific adhesion–receptor interactions of *H. pylori* vesicles

**Fig. 6:**
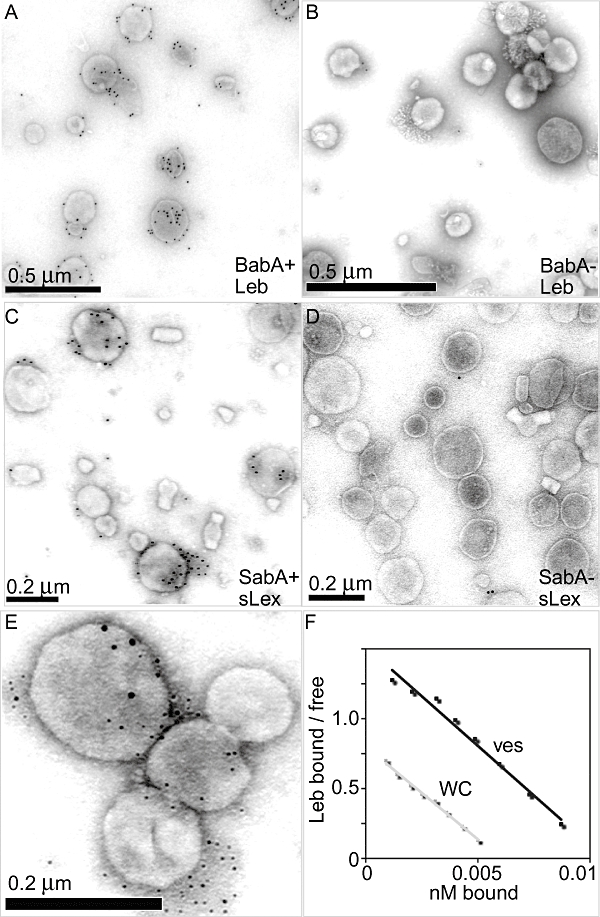
Presence of receptor-binding adhesins on the surface of *H. pylori* vesicles. A. Electron microscopy was used to detect the binding of biotinylated Leb–receptor conjugates and streptavidin-conjugated gold particles to BabA adhesins on the surface of *H. pylori* vesicles isolated from strain 17875/Leb. B. Only background labelling was observed on vesicles isolated from strain 17875/sLex that is devoid of BabA expression. Bar length in A and B represents 0.5 µm. C. sLex-binding adhesins were identified on 17875/sLex-derived vesicles using sLex–receptor conjugate and streptavidin-conjugated gold particles. D. Only background labelling was observed on vesicles isolated from strain 17875/Leb, which lacks sLex-binding activity. Bar length in C and D represents 0.2 µm. E. Electron micrographs of vesicles incubated with anti-BabA antibodies detected by 10 nm gold particles and sLex receptor conjugate detected by 5 nm gold particles. The larger 10 nm dots correspond to BabA adhesins and the 5 nm dots represent SabA adhesins. F. A receptor displacement assay according to the Scatchard method was used to measure the affinity constant (*K*_a_) of ^125^I-Leb receptor conjugate to the BabA adhesin in *H. pylori* whole cells (WC) (*K*_a_ = 1.34E + 11) versus vesicles (ves) (*K*_a_ = 1.42E + 11) from strain 17875/Leb.

To analyse if the BabA and the SabA adhesins are present on the vesicle surface and also their ability to bind their cognate receptors, a combination of streptavidin conjugated gold particles and receptor conjugates were used for visualization of binding by electron microscopy. For these studies, three *H. pylori* strains were included: strain CCUG17875, which expresses both BabA and SabA adhesins, a variant clone, 17875/Leb, which only has Leb-binding properties, and strain 17875/sLex, which expresses the SabA adhesin but has the *babA1* and *babA2* genes knocked-out. Vesicles from strains 17875/Leb (BabA+/SabA−) and 17875/sLex (BabA−/SabA+) were probed with biotinylated Leb/sLex receptor conjugates and bound conjugate was detected with streptavidin conjugated gold particles (Fig. 6). Gold particles were distributed over the surface of BabA+ vesicles isolated from 17875/Leb when incubated with the Leb–receptor conjugate (Fig. 6A). In contrast, only a few gold particles were detected on vesicles isolated from the Leb-non-binding mutant strain, 17875/sLex (Fig. 6B). When vesicles isolated from strain 17875/sLex (SabA+) were probed with the sLex conjugate, gold particles were detected on the surface of SabA+ vesicles (Fig. 6C). Vesicles isolated from strain CCUG17875 (BabA+/SabA+) were subjected to double-staining using BabA antibodies and the sLex–receptor conjugate in combination with gold particles of 10 and 5 nm in size respectively. Thus, one and the same vesicle can exhibit both Leb and sLex-binding properties (Figs 6E and S5).

*Helicobacter pylori* vesicles separated according to their size were also analysed for the presence of Leb-binding adhesins. In general, it appeared that larger vesicles typically carried more Leb-binding BabA adhesins than smaller *H. pylori* vesicles (Fig. 3A–C). Background staining was more commonly observed in samples containing smaller vesicles, which we hypothesize to be staining that corresponds with the presence of soluble proteins identified in fractions 1 and 2 (Fig. S1). Gold particles corresponding to Leb-binding of BabA adhesins visible on electron micrographs from fractions 2, 4 and 9 were counted. The proportion of Leb-binding vesicles present in each fraction correlated with the number of larger vesicles present in each fraction. For example, fraction 2 contained 23% of Leb-binding vesicles, fraction 4 contained 60% of Leb-binding vesicles, and fraction 9 contained 81% of Leb-binding vesicles (Fig. 3E).

Further biochemical characterization of the vesicle BabA–Leb-binding properties was performed using a modified Scatchard method (Aspholm *et al*., 2006b) (described in *Experimental procedures*) to measure the affinity. The affinities of whole 17875/Leb bacterial cells versus vesicles isolated from the same strain were similar (Fig. 6F). Whole bacterial cells of strain 17875/Leb exhibited an affinity for Leb of *K*_a_ = 1.34E + 11, while the affinity of vesicles for Leb was *K*_a_ = 1.42E + 11. The observation that the BabA adhesin of *H. pylori* vesicles binds to the Leb–receptor with the same affinity as whole bacterial cells suggests that the adhesins presented by *H. pylori* vesicles exhibit a similar folding as the adhesins presented on whole bacterial cells.

### Properties for intimate adherence of *H. pylori* vesicles to human gastric mucosa

**Fig. 7:**
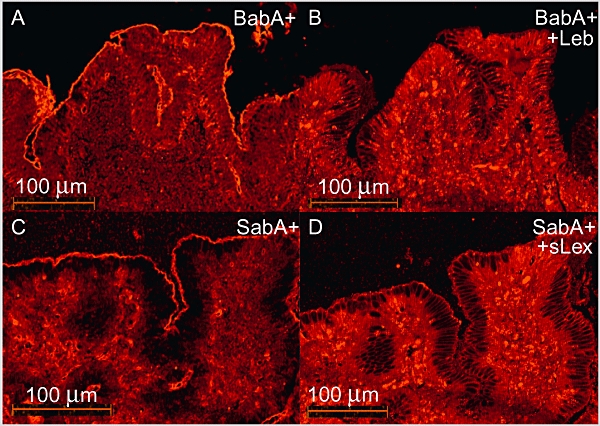
Adherence of *H. pylori* vesicles to human gastric epithelium. Vesicle adherence to the epithelium of human gastric mucosa via BabA–Leb interactions and SabA–sLex interactions was detected using biotinylated anti-*H. pylori* antibody and streptavidin-conjugated Cy3. Vesicles isolated from *H. pylori* strains 17875/Leb (BabA+/SabA−) and 17875/sLex (BabA−/SabA+) were incubated with human gastric tissue sections as follows: A. 17875/Leb vesicles. B. 17875/Leb vesicles pre-incubated with Leb receptor conjugate prior to incubation with gastric tissue sections. C. 17875/sLex vesicles. D. 17875/sLex vesicles pre-incubated with sLex receptor conjugate prior to incubation with gastric tissue sections. Bar lengths represents 100 µm.

*Helicobacter pylori* vesicles have previously been identified to attach to and to be taken up by human epithelial cells (Fiocca *et al*., 1999; Heczko *et al*., 2000); however, details in the adherence properties of *H. pylori* vesicles have remained elusive. Vesicles from strain 17875/Leb (BabA+/SabA−) and 17875/sLex (BabA−/SabA+) were analysed for binding *in vitro* to human gastric mucosa. Histo-tissue sections were probed with vesicles and bound vesicles were identified with antibodies against *H. pylori*. Both BabA (Figs 7A and S6) and SabA (Figs 7C and S6) mediated binding of vesicles to the gastric epithelial lining. To study the adhesion–receptor specificity operating in the vesicle–tissue interaction in more detail, vesicles were pre-incubated with receptor conjugate prior to incubation with the gastric tissue sections. Pre-incubation of 17875/Leb vesicles with Leb–receptor conjugate blocked BabA–Leb mediated adherence demonstrating the role of the BabA adhesins in mediating *H. pylori* binding (Figs 7B and S6). In contrast, pre-incubation of 17875/Leb vesicles with sLex–receptor conjugate did not affect adherence (data not shown). However, pre-incubation of 17875/sLex vesicles with sLex receptor conjugate did abolish adherence to the gastric mucosa (Figs 7D and S6), while pre-incubation with Leb did not affect adherence (data not shown). Thus, *H. pylori* vesicles carries biologically active, fully folded BabA and SabA adhesins on the surface, which can mediate specific interactions to receptors present in human gastric mucosa.

### CagA is associated with the surface of *H. pylori* vesicles

**Fig. 8:**
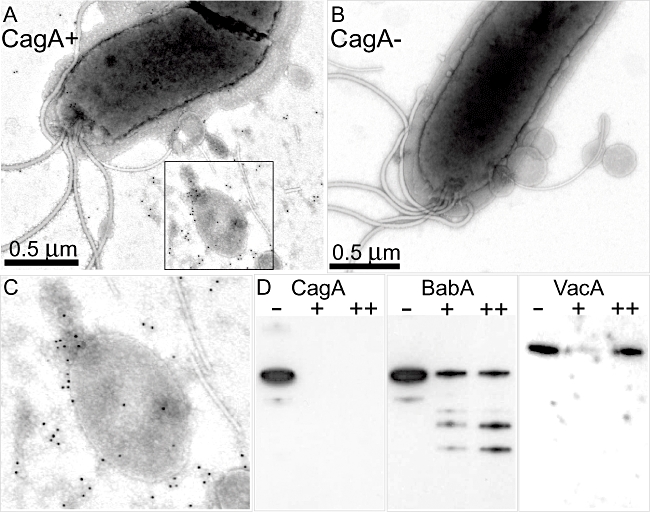
CagA is localized to the surface of *H. pylori* vesicles. Immunogold-labelling of CagA protein. A. Electron micrographs of *H. pylori* strain P12. B. Electron micrographs of *H. pylori* strain P12Δ*cagA*. Bar length in A and B represents 0.5 µm. C. An enlargement of the indicated area in (A). D. Vesicles of strain P12 treated with Trypsin (+), NP40 (++) or neither (-) as indicated. Samples were analysed by immunoblot using antibodies against BabA, CagA and VacA respectively.

Biochemical analysis identified CagA as a protein component of *H. pylori* vesicles. Immunogold analysis localized CagA to the bacterial cell surface, vesicle surface (Fig. 8C), and surroundings of strain P12 (Fig. 8A). In contrast, strain P12Δ*cagA* was negative for CagA staining as expected (Fig. 8B). The association of CagA with the vesicle surface was further confirmed using a protease assay. *H. pylori* P12 vesicles were digested with trypsin in the presence or absence of the detergent, NP40, and then analysed by immunoblotting for the presence of CagA, BabA and VacA proteins (Fig. 8D). Protease treatment degraded CagA completely, while BabA was only partially degraded. Based on these results, we hypothesize that CagA is associated with the surface of *H. pylori* vesicles, in contrast to BabA that is an OM protein and partly integrated into the vesicle membrane. VacA was completely degraded by protease treatment in the absence of detergent, although in the presence of detergent VacA was only partially degraded.

## Discussion

Although the shedding of vesicles by Gram-negative bacteria was described several decades ago, the more recent finding that vesicles shed from pathogenic bacteria can act as vehicles for delivery of host–effector molecules and toxins is of particular interest. Therefore, the vesicles of disease-associated bacteria have significant implications for the pathogenesis of bacterial disease. Several studies have focused on the delivery of toxins, particularly *E. coli* toxins such as LT toxin, ClyA, CNF1 toxin and α-haemolysin (Horstman and Kuehn, 2000; Wai *et al*., 2003; Balsalobre *et al*., 2006; Kouokam *et al*., 2006). In spite of detailed biochemical and functional analyses of vesicles from a multitude of different species, the role of bacterial vesicles in disease-associated pathogenesis is still far from being clear. Here we studied vesicles of the gastric pathogen, *H. pylori*, using detailed biochemical assays. Major protein and phospholipid components were identified and evaluated. Selected vesicle proteins were characterized in more detail and in particular adherence proteins and their role in specific mechanisms of vesicle–host interactions.

The detailed mechanisms of vesicle formation are not fully defined. However, one approach to further understanding of the shedding mechanism and its potential biological impact is to identify the composition of vesicles. Samples of crude and gradient-purified vesicle fractions were analysed by a combination of electron microscopy and SDS-PAGE. These analyses demonstrated that density centrifugation was sufficient to remove cell debris, broken flagella and soluble proteins that contaminated crude vesicle fractions. To identify major vesicle protein components, nanoflow LC FT-ICR MS/MS was used. Bacterial vesicles have been described to contain predominantly OM proteins and periplasmic components; however, LPS, cytoplasmic components, DNA and RNA have also been found to be present (Horstman and Kuehn, 2000; Wai *et al*., 2003; Post *et al*., 2005; Nevot *et al*., 2006). We specifically identified peptides corresponding to more than 300 different *H. pylori* proteins from the 34 bands that were cut out from our vesicle sample. A majority (77%) of all *H. pylori* OM proteins were found in the vesicles. Despite the ability of MS to identify peptides present in small amounts, the method is not quantitative. Therefore, as seen upon comparison of protein profile of OM fractions and vesicles on SDS-PAGE, the OM proteins present in the vesicles are in greater abundance than other proteins, as described for vesicles previously. Quantification of a series of proteins present in the OM and the vesicles show a clear distinction in the relative levels, which further shows that protein composition of vesicles is clearly distinct from the OM as well as intact *H. pylori* cells. The OM proteins represent only 16% of the total number of proteins that we identified in the vesicles. In addition, and in accordance with our results and that of other laboratories recently summarized by Lee *et al*. (2008), it is evident that cytoplasmic proteins are naturally occurring components in bacterial vesicles and possibly more abundant than previously appreciated. The presence of cytoplasmic components can in part be explained by the choice of experimental assays. Sensitive MS methods, such as LC FT-ICR MS/MS, can detect even small amounts of complex protein compositions in a gel sample and thereby provide a more comprehensive analysis. In addition, we probed vesicles with the IM marker ComB10 in immunoblots and indeed obtained a signal (data not shown). Thus, the suggested mechanisms for shedding of vesicles may not be complete since they do not include how IM and cytoplasmic proteins are trapped within the vesicle lumen (Kuehn and Kesty, 2005; Mashburn-Warren and Whiteley, 2006; Deatherage *et al*., 2009).

The results of a proteome analysis are dependent on the type of sample analysed. Environmental conditions such as growth phase and growth medium affect gene expression, which then will affect the composition of vesicles produced. This is illustrated by the results of the protein profiles of broth-derived vesicles versus agar-derived vesicles that differed significantly. In an MS analysis of *E. coli* vesicles, vesicles of 20–40 nm were predominant (Lee *et al*., 2007). An alternative selection of vesicle samples would probably have given a somewhat different outcome of the proteomic analysis. Therefore, it is important that the results of an MS analysis are associated with the specific conditions of the samples analysed and not extrapolated to be a generalized conclusion. The MS analysis in this study was based on the 34 most prominent bands from an *H. pylori* sample corresponding to growth on blood agar plates *in vitro*, although a more ideal sample would have been vesicles derived from biopsy material that would represent the *in vivo* expression pattern of proteins in *H. pylori* vesicles. Such protein profile would provide key factors to unravel the complex biogenesis of vesicles.

To reliably define the phospholipid composition of *H. pylori* membranes, we used liquid-state ^31^P,^1^H NMR. The composition of whole *H. pylori* cells, OM, IM and vesicles was all dominated by PE and CL. These results are consistent with studies of coccoid *H. pylori* cells (Shimomura *et al*., 2004). The main phospholipids present in vesicles differ between bacterial species. In vesicles of *N. meningitidis*, PE and PG were most abundant (Post *et al*., 2005). In these studies and previous studies of coccoid *H. pylori* (Shimomura *et al*., 2004), significant amounts of LPE were detected in all membrane fractions, although a smaller amount was detected in IM fractions. Both lysophospholipids and CL have been associated with increased membrane curvature (Huang *et al*., 2006). Thus, the presence of lysophospholipids and CL in bacterial vesicles is hypothesized to be essential to achieve the high degree of curvature associated with these smaller vesicles. Alternatively, formation of lysophospholipids might also be a sign of bacteria being under stress and may play a role in destabilizing *H. pylori* membranes (Tannaes *et al*., 2000; 2001;). In studies by Tannaes and co-workers a relationship between higher levels of lysophospholipid present and ulcer disease was identified (Tannaes *et al*., 2005). Future studies of lysophospholipid content in vesicles from strains of bacteria associated with various levels of peptic ulcer disease may provide additional insight into the possible role of lysophospholipids in destabilizing membranes to facilitate uptake by the host to affect disease outcome.

Adherence is an important step in the delivery of toxins and other effector molecules by bacteria to target tissues. So far, only few detailed mechanisms of the interactions between bacterial vesicles and host cells have been described. To our knowledge, this study is the first to describe the biological activity and biochemical characterization of adhesins present on bacterial vesicles. MS analysis identified both the BabA and SabA adhesins as components of *H. pylori* vesicles, which was further confirmed in immunoblotting studies. Using receptor conjugates and electron microscopy, we could demonstrate receptor-binding activity of both the BabA and the SabA adhesin. The binding specificity of both adhesins was as high as for whole bacterial cells (Ilver *et al*., 1998; Mahdavi *et al*., 2002), and binding to gastric histo-tissue sections was completely inhibited by pre-incubation with cognate receptor conjugate prior to addition to the sections. Biochemical analysis of BabA–Leb interactions revealed that the Leb-binding affinity for vesicles was almost identical to that of whole bacterial cells. This suggests that BabA is functionally equivalent in vesicles and whole bacterial cells, and that BabA proteins are displayed similar on both vesicles and whole bacterial cells.

Differences in size and density (i.e. protein/lipid ratio) observed for vesicles reflect differences in vesicle composition. When vesicle samples that differed in density were compared in two separate MS analyses, the MS II analysis of 32 bands from vesicles with a density of 1.17–1.18 g ml^−1^ identified 126 proteins, 93% of which overlapped with proteins identified in the MS I analysis of vesicles with densities of 1.15–1.20 g ml^−1^. The higher density vesicles, probably containing a higher protein/lipid ratio, therefore correlated with a greater number of proteins identified. Differences in protein content between vesicles and the impact of these differences in biological activity as here illustrated in terms of receptor binding were clearly illustrated by the higher prevalence of Leb-binding BabA adhesins identified in the larger vesicles. Balsalobre and co-workers have also found differences in the composition in *E. coli* vesicles, with α-haemolysin associated with larger vesicles (Balsalobre *et al*., 2006). However, not all *H. pylori* vesicles analysed carried Leb-binding or sLex-binding adhesins. Similar observations of differences in vesicle phenotypes were reported by Kesty and Kuehn (2004). In these studies, only vesicles carrying the OM Ail adhesin/invasin were taken up by host cells and not vesicles devoid of the Ail protein. In addition, LPS has been described to affect shedding of bacterial vesicles and possibly also the size of the shedded vesicles (Kadurugamuwa and Beveridge, 1998; Nguyen *et al*., 2003; Mashburn-Warren and Whiteley, 2006).

Although the role of bacterial vesicles in pathogenesis is not fully understood, some characteristics have been established. For example, delivery of host–effector molecules and toxins has been demonstrated, and a predatory role for these factors has been identified based on the presence of anti-bacterial factors such as auto-lysin detected in vesicles of *Pseudomonas aeruginosa*. Auto-lysin has been shown to effect the degradation of cell walls of both Gram-positive and Gram-negative bacteria (Li *et al*., 1996). Moreover, Tan and co-workers have demonstrated that *Moraxella catarrhalis* vesicles carrying the UspA1/A2 proteins contribute to serum resistance and increased survival of co-infecting *Haemophilus influenzae* (Tan *et al*., 2007). Vesicles of *M. catarrhalis* have also been shown to act as decoys in B-cell interaction and redirect the adaptive humoral immune response by avoiding direct contact with the bacteria (Vidakovics *et al*., 2010). Similarly, *Neisseria gonorrhoeae* has also been shown to protect itself from bactericidal factors via vesicles elaborated from serum-resistant strains. Such vesicles were found to have an inhibitory effect on the destruction caused by normal human serum (Pettit and Judd, 1992).

In addition to the BabA and SabA adhesins, we also identified other virulence-associated factors including the urease subunits, OipA and the CagA protein. We confirmed that the VacA toxin can be associated with *H. pylori* vesicles, consistent with the results of other studies (Fiocca *et al*., 1999; Ricci *et al*., 2005). The effector and oncoprotein, CagA, has been described to induce changes in cell morphology, cell motility, differentiation and polarity (Hatakeyama, 2008). Using immunogold electron microscopy, we demonstrated that CagA is present on the surface of vesicles. Further analysis using a protease assay suggested that CagA is associated to the surface of vesicles. This is in contrast with the protease assay results for the BabA adhesin where trypsin digestion was incomplete even in the presence of detergents, supporting that BabA is an integral component of the OM. In the presence of detergent trypsin could only partially degrade VacA. Delivery of VacA by vesicles induces a vacuolating phenotype, although with lower vacuolating activity then that mediated by whole bacteria (Ricci *et al*., 2005), which suggest differences in oligomerization. One interpretation of our result is that the detergent induces oligomerization of VacA and thereby the trypsin cleavage sites are hidden.

Injection of CagA by bacteria is dependent on the T4S component, CagL, and its interaction with β1-integrins (Kwok *et al*., 2007). In our studies we identified several proteins encoded by the *cag*PAI. With histo-sections from human gastric epithelium, we showed here that both the BabA and SabA adhesins on the vesicle surface mediated specific receptor interactions with human gastric tissue. Clearly, an exciting possibility is that delivery of CagA to gastric host cells might be mediated by *H. pylori* vesicles and thus may occur independently of CagL or a complete T4S system. Uptake of vesicles by membrane fusion has been described for *P. aeruginosa* and uptake via endocytosis of *E. coli* vesicles (Kesty *et al*., 2004; Bomberger *et al*., 2009) and in accord delivery of virulence factors. Further analysis of pathways for uptake of *H. pylori* vesicles and delivery of host–effector proteins, such as CagA, are currently explored in our laboratory.

The small genome of *H. pylori* requires the bacterium to be dependent on nutrient leakage from host cells. Adherence facilitates access of *H. pylori* to nutrients, while at the same time promotes delivery of bacterial toxins and host–effector molecules. In such perspective, adherence may be devastating due to the vigour of bactericidal host responses. The persistent, often lifelong infections caused by *H. pylori* probably result from a balanced relationship between the needs of *H. pylori* and the defences of the host. It is therefore possible that direct contact of vesicles with host cells may allow the bacteria to stay in a safe distance from host cell cytotoxicity and still achieve the release of nutrients. Thus, shedding of vesicles filled with toxins, effector molecules and other factors may interfere with the host cell and/or host immune responses, thereby affecting the persistence of infection. Further studies are needed to more fully characterize the interactions between *H. pylori* vesicles and host cells, particularly in regard to gastric tissue; however, valuable insights have been provided by this study.

## Experimental procedures

### Bacterial strains and growth conditions

Bacterial strains used in this study: CCUG17875 (CCUG, Gothenburg, Sweden); 17875/Leb (Ilver *et al*., 1998); 17875Δ*babA1*::kanΔ*babA2*::cam, here called 17875/sLex (Mahdavi *et al*., 2002); P12 (Odenbreit *et al*., 1996); P12Δ*cagA* (Backert *et al*., 2001) and P12Δ*cagPAI* (Backert *et al*., 2005). Bacteria were grown on blood agar plates of Brucella agar supplemented with 10% bovine blood (Svenska Labfab, Sweden), 1% IsoVitox Enrichment (Dalynn Biologicals), and an antibiotic mixture of amphotericin B (4 mg l^−1^), vancomycin (10 mg l^−1^) and trimetoprim (5 mg l^−1^). When needed, the agar was supplemented with chloramphenicol (20 mg l^−1^) and/or kanamycin (25 mg l^−1^). Bacteria were also grown in Brucella broth supplemented with 10% calf serum (Gibco), 1% IsoVitox Enrichment and the same antibiotic mix described above. *H. pylori* were routinely grown at 37°C in a microaerophilic environment of 5% O_2_, 10% CO_2_ and 85% N_2_.

### Isolation of *H. pylori* vesicles

Isolation of *H. pylori* vesicles was performed according to Wai *et al*. (2003) with the following modifications: *H. pylori* were grown on blood agar plates for 2 days, harvested in 20 mM Tris-HCl (pH 8.0), and centrifuged twice for 30 min at 8000 *g* at 4°C. Alternatively, *H. pylori* were grown in broth culture to early stationary phase and harvested by centrifugation for 30 min at 4000 *g* at 4°C. The supernatant was filtered through a 0.22 µm cellulose acetate filter and centrifugated for 3 h at 150 000 *g* at 4°C. The pellet containing vesicles was resuspended in 20 mM Tris-HCl (pH 8.0). To purify vesicles according to density, vesicles were subjected to flotation in a continuous Histodenz gradient [20–60% Histodenz (Sigma) in 250 mM sucrose/20 mM Tris-HCl (pH 8.0)] and separated by equilibrium centrifugation for 16 h at 200 000 *g* at 4°C (Lundmark and Carlsson, 2003). For separation of vesicles according to size, crude vesicle preparations were loaded on top of a 5–15% continuous Histodenz gradient and centrifuged for 1.5 h at 150 000 *g* at 4°C. Vesicles were purified from the Histodenz using a 4× dilution followed by ultra-centrifugation for 90 min at 120 000 *g* at 4°C. The pellet fraction was resuspended in 20 mM Tris-HCl (pH 8.0).

Average vesicle size was calculated by measuring their diameter in electron micrographs representing 207 vesicles (fraction 2); 146 (fraction 4); 188 (fraction 9).

### Isolation of outer membranes

For the NMR analysis, total membrane samples were separated into outer and inner membrane fractions using sucrose density gradients and centrifugation as described by Horstman and Kuehn (2000). Briefly, *H. pylori* cells were harvested and washed in 20 mM Tris-HCl (pH 7.5) and centrifuged at 3000 *g* for 10 min. Frozen cells were lysed with X-press. Unbroken cells were removed by centrifugation at 3000 *g* for 10 min. The supernatant was applied to a sucrose cushion [2 ml 55% sucrose, 0.5 ml 5% sucrose in 20 mM Tris-HCl (pH 7.5)/0.5 mM EDTA]. Membranes were removed from the interface with a syringe needle (19 gauge) and diluted to 2.5 ml in 20 mM Tris-HCl (pH 7.5)/0.5 mM EDTA. Diluted membranes were loaded on a stepwise sucrose gradient (0.4 ml 60%, 0.9 ml 55%, 2.2 ml 50%, 2.2 ml 45%, 2.2 ml 40%, 1.3 ml 35% and 0.4 ml 30%) and centrifuged at 150 000 *g* for 18 h. Fractions were collected from the bottom in the same volumes described for the composition of the stepwise gradient.

For immunoblots in Fig. 5D, total membrane was separated into outer and inner membrane fractions using detergent for comparison of protein components in OM and vesicles. Briefly, total membranes were isolated as above and incubated 30 min in 2% *N*-laurylsarcosyl at room temperature (RT). OM proteins were collected by centrifugation for 30 min at 50 000 *g* at 4°C.

### Isolation of lipids

Lipids were isolated essentially as previously described (Hamilton *et al*., 1992) using methanol/chloroform extractions. Lipids were solubilized in deuterated chloroform : methanol (2:1). To avoid paramagnetic contamination, CsEDTA was added to a final concentration of 10 mM according to Meneses and Glonek (1988).

### NMR analysis

High-resolution NMR spectra were recorded on a DRX600 NMR spectrometer (Bruker, Fällanden, Switzerland) equipped with a ^1^H, ^13^C, ^31^P cryo-probe with z-gradient. NMR spectra were processed using TopSpin Software 2.0 (Bruker). The amount of lipid sample ranged from 0.5 to 10 mg, with corresponding detection limits between 2% and 0.1% of an individual phospholipid respectively. Two-dimensional ^31^P,^1^H correlation spectra were used to unambiguously assign phospholipids based on their ^31^P and ^1^H chemical shifts, and ^31^P,^1^H J couplings. Using one-dimensional projections of the 2D spectra, the relative amount of each phospholipid was estimated by integration of the peak area (Petzold *et al*., 2009). To calculate the concentration of cholesterol, 2D ^13^C,^1^H correlations were recorded according to (Sattler *et al*., 1999). For this purpose, integrals of well-resolved C-H groups of cholesterol and PG were compared.

### SDS-PAGE and immunoblot analysis

Protein samples were separated on 4–12% Bis-Tris gel (Invitrogen) or 10% or 15% gels and stained with Commassie Page Blue (Fermentas). BCA Protein Assay Kit (Pierce) or Quibit fluorometeter (Invitrogen) was used for measurements of protein concentration. Protein extracts contain 2% SDS to eliminate any interference from lipids.

Immunoblots were performed essentially as previously described by Bäckström *et al*. (2004). Primary antibodies included: anti-BabA (AK277) (Odenbreit *et al*., 2002b); anti-SabA (AK278) (Odenbreit *et al*., 2002b); anti-CagA (Austral Biologicals); anti-VacA (AK204) (Fischer *et al*., 2001); anti-AlpB (AK262) (Odenbreit *et al*., 2002a); anti-OipA (AK282) (Kudo *et al*., 2004); anti-ComB10 (AK252) (Hofreuter *et al*., 2001); anti-CagT; anti-CagX; anti-CagY; anti-CagM; anti-CagN; anti-VirD4. Secondary antibody: anti-rabbit horseradish peroxidase (DakoCytomation). Membranes were developed using Supersignal West Pico Chemiluminescence Substrate (Pierce) and signals were recorded using X-ray film or scanning using Kodak Molecular Imaging Software 4.0.3.

### Quantification of vesicle proteins

Quantification of specific proteins in OM and vesicle samples was performed with immunoblot analysis. Series of 1, 2 and 4 µg protein from each sample, respectively, were loaded on SDS-PAGE gels and stained to verify the correct amount of loaded samples. Next, samples were separated by SDS-PAGE and blotted on to PVDF membranes and probed with antibodies. The densities of signals in the serial dilutions for each sample were measured with the Image J program (http://rsbweb.nih.gov/ij/). Linear regression was applied to data from three-four experiment sets of immunoblots to establish the density for each protein prior to calculation of the relative ratio of OM and vesicles.

### In-gel protein digestion

In-gel protein digestion with trypsin was performed as described by Shevchenko *et al*. (1996) with some minor modifications. Briefly, gel pieces were destained by washing three times in 25 mM NH_4_HCO_3_/50% CH_3_CN and one time in 25 mM NH_4_HCO_3_/50% CH_3_OH. Gel pieces were dried in a vacuum centrifuge and incubated with digestion buffer (50 mM NH_4_HCO_3_, 10 ng µl^−1^ trypsin) at 37°C overnight. Peptides were extracted in 50% CH_3_CN/1% CH_3_COOH and the supernatant evaporated in a vacuum centrifuge. Prior to MS analysis, the peptides were reconstituted in 0.2% HCOOH.

### Nanoflow LC FT-ICR MS/MS and database searches

Sample injections were made with an HTC-PAL autosampler (CTC Analytics AG, Zwingen, Switzerland) connected to an Agilent 1100 binary pump (Agilent Technologies, Palo Alto, CA, USA). Peptides were trapped on a pre-column and separated on a reversed phase column. The nanoflow LC-MS/MS was performed on a hybrid linear ion trap-FT-ICR mass spectrometer equipped with a 7T ICR magnet (LTQ-FT, Thermo Electron, Bremen, Germany). The spectrometer was operated in data-dependent mode that automatically switched to MS/MS mode. MS spectra were acquired in the FT-ICR, while MS/MS spectra were acquired in the LTQ trap. For each scan of FT-ICR, the three most intense ions, doubly or triply charged, were sequentially fragmented in the linear trap by collision-induced dissociation. All the tandem mass spectra obtained were submitted to MASCOT (Matrix Science, London) for comparisons against all species in the NCBI database, MS I; NCBInr 080130 (5 878 816 sequences), MS II; NCBInr 060802 (3 841 279 sequences). The search parameters included MS accuracy of 5 p.p.m., MS/MS accuracy of 0.5 Da, one missed cleavage by trypsin was allowed, fixed propionamide modification of cysteine, and variable modification of oxidized methionine. For protein identification, the minimum criteria were, one tryptic peptide match at or above the 99% level of confidence (MOWSE score > 45) and an additional peptide match at the 95% level (MOWSE score > 30). Strain CCUG17875 used in the MS analysis is not genome sequenced. Therefore, when multiple proteins matched the same set of peptides, we report Gene bank accession number from *H. pylori* strains in the following order; 26695, J99, HPAG1 and other *H. pylori* strains. Strain 26695 was chosen as a reference strain for gene numbers that are presented in Table S1. For homologous proteins, with different scores, a sequence distance over 90% (MegAlign, DNA star), was required. The Gene bank accession number with the highest MOWSE score is presented. The biological function of identified proteins was assigned by COG classification, Clusters of Orthologous Groups of proteins (http://www.ncbi.nlm.nih.gov/COG). *H. pylori* OM proteins, defined by Alm *et al*. (Alm *et al*., 2000), were sorted into a separate class. Sequence coverage was calculated in per cent by dividing the number of amino acids in matched peptides with the total number of amino acids in the identified protein. Twenty-six hits against human and cow proteins were deleted.

### Receptor affinity replacement assay

Scatchard analysis was performed as previously described (Aspholm *et al*., 2006b). Briefly, solutions with 0.2 ng ^125^I-labelled Leb receptor glyco-conjugate and unlabelled conjugate (HSA-Leb) (Isosep AB, Tullinge, Sweden) were added in a dilution series from 0 to 4 ng to eight samples of whole cells and vesicles respectively. A Leb-non-binding strain was added to the samples as a carrier to facilitate pelleting of the vesicles. The pellet and supernatant of the vesicles were separated after centrifugation for 1 h at 42 000 *g* at 4°C, while the whole-cell samples were centrifugated for 10 min at 16 000 *g*. Affinity (*K*_a_) corresponds to the slope of the respective curve.

### Electron microscopy analyses

Samples of whole-cell *H. pylori* and isolated vesicle samples were applied to formvar-coated copper grids, negatively stained with 1% sodium silicotungstate, and analysed with a JEOL 1230 transmission electron microscope. Immunogold analysis was performed as described in Bäckström *et al*. (2004) using biotinylated-Leb or biotinylated-sLex, detected with 10 nm gold-anti-biotin (GAB10: British Biocell International, Cardiff, UK). Anti-CagA antibodies were detected with 10 nm gold-anti-rabbit particles (GAR10: British Biocell International). Double-staining was performed using anti-BabA antibodies and 10 nm gold particles (GAR10) in combination with biotinylated-sLex and 5 nm gold particles (GAB5). Calculations of Leb-binding vesicles were based on a series of electron micrographs representing in total 524 vesicles (fraction 2), 351 (fraction 4) and 183 (fraction 9).

### Adherence to gastric tissue sections

Human gastric tissues were deparaffinized and blocked in blocking buffer [1× PBS (5 mM KH_2_PO_4_/20 mM K_2_HPO_4_/85 mM NaCl)/0.05% Tween-20/1% BSA] at RT for 1 h*. H. pylori* vesicles purified by a Histodenz gradient were added to the tissue sections and incubated for 2 h. Alternatively, vesicles were pre-incubated with Leb or sLex receptor conjugate for 2 h prior to addition to tissue sections. Tissue sections were washed in washing buffer (1× PBS/0.05% Tween-20), blocked with blocking buffer for 20 min, then incubated with biotinylated anti-*H. pylori* antibody (Abcam, Cambridge, UK) overnight at 4°C and thereafter for 1 h at RT. Tissue sections were subsequently washed and incubated with streptavidin-Cy3 (Amersham Bioscience) for 1 h. Cy3-fluorescence on tissue sections was analysed using a Zeiss AxioImager Z1 microscope, apotome and Axiovision software v4.5. Alternatively, biotinylated anti-*H. pylori* antibody was detected with streptavidin-ABComplex (DAKO A/S) for 1 h and DAB+ (Carpinteria, CA, USA) followed by counterstaining with Mayers haematoxylin.

### Trypsin digestion of vesicles

Purified P12 vesicles were subjected to trypsin digestion (10%) for 1 h on ice either in the presence or absence of 1% NP40 (Thermo Scientific, Waltham, USA). Samples were subsequently resuspended in SDS-PAGE sample buffer, separated by SDS-PAGE, and proteins detected in immunoblots using anti-CagA, anti-VacA and anti-BabA antibodies.
