# Supplementary material for: Biochemical and functional characterization of Helicobacter pylori vesicles
Source: Mol Microbiol. 2010 Aug 5;77(6):1539–55. doi: 10.1111/j.1365-2958.2010.07307.x (PMC3068288; doi:10.1111/j.1365-2958.2010.07307.x)
Supplement: Supplementary file 1 [file mmi0077-1539-SD1.pdf]

Fig. S1

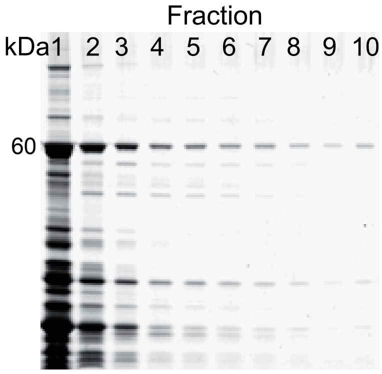

SDS-PAGE analysis of vesicles separated according to size
Crude vesicle preparations were separated according to size.
The same volume from fractions 1-10 was analyzed by SDS-PAGE.
Fraction 1 corresponds to the top fraction.

Fig. S2

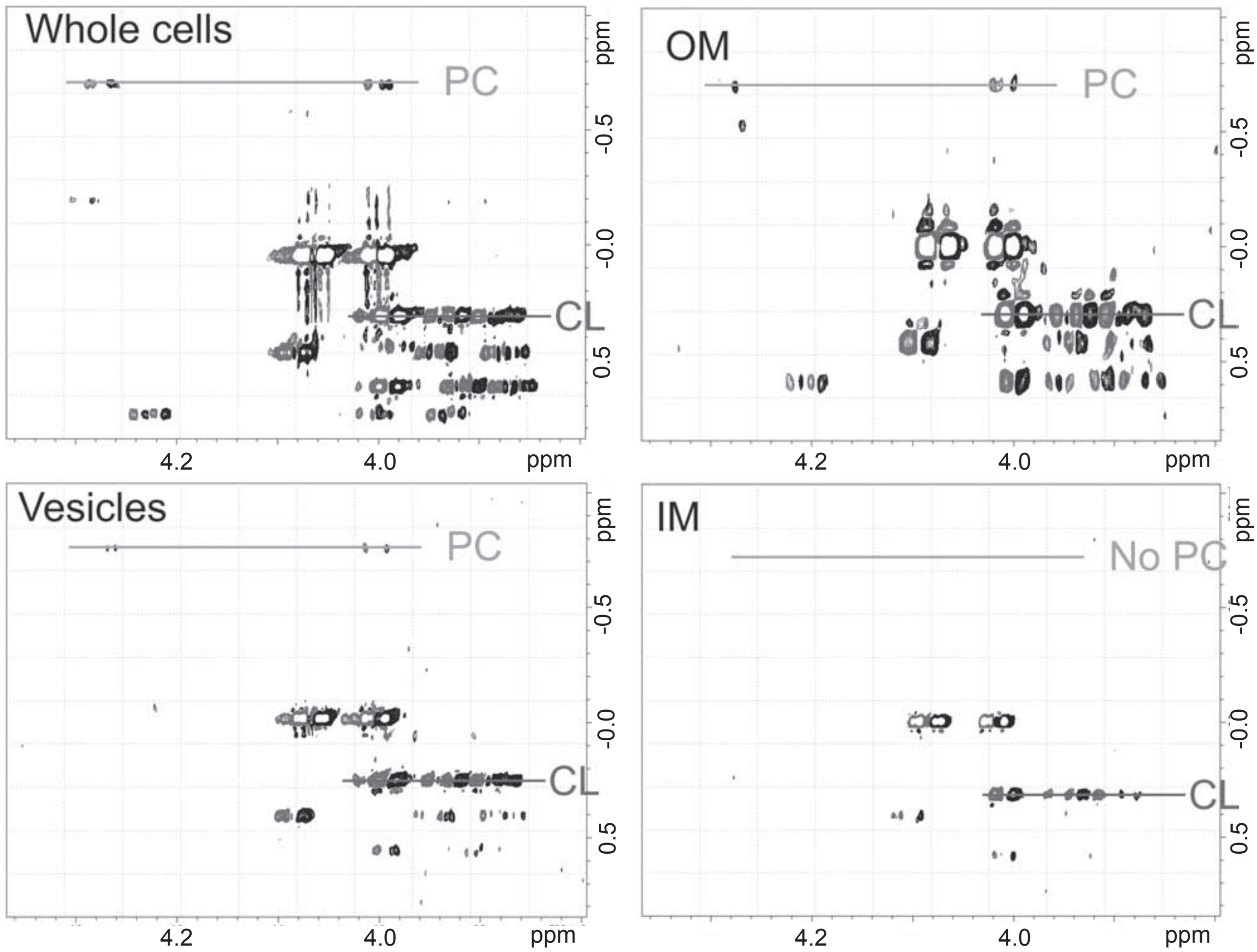

2D 31P, 1H NMR correlation spectra
H. pylori bacterial samples from whole cells, vesicles outer membrane, and inner membrane analyzed for phospholipid composition.
Signals from phophatidylcholine (PC) and cardiolipin (CL) are marked, showing the absence of PC in IM and the clear CL signal, indicating that the shift of CL in the 1D spectrum is a result of the solvents used.

Relative concentration of phospholipids found in isolated *H. pylori* samples

	LPG	CL	PE	LPE	PG	LPC	PC
whole cells	0.7	9.6	70.4	7.4	10	0.3	1.5
vesicles	1.2	18.1	67.7	8.6	3.2	0.3	0.9
Outer membrane	ND	15.5	62.7	9.8	8.5	1.4	2.1
Inner membrane	ND	15.8	72.2	6.4	6.2	ND	ND

ND = not detectable

Table S1: MS I - *H. pylori* vesicle proteins

Gene bank accession number	Protein name	Gene no.	Mass (kDa)	Mowse score	No. of peptides	Sequence coverage (%)	MSII
**OUTER MEMBRANE PROTEINS**							
**Major outer membrane protein family**							
NP_207704	Outer membrane protein AlpA, HopC (omp20)	HP0912	56,1	4744	206	29	
CAB05387	Outer membrane protein AlpB, HopB (omp21)	HP0913	55,5	2866	191	32	x
AAC38081	Outer membrane protein BabA, HopS (omp28)	HP1243	80,9	15959	792	49	x
AAB97730	Outer membrane protein BabB, HopT (omp19)	HP0896	77,7	6924	215	27	
AAQ57665	Outer membrane protein OipA, HopH (omp13)	HP0638	34,2	1383	81	35	x
YP_627450	Outer membrane protein SabA, HopP (omp17)	HP0725	72,1	2760	221	29	x
NP_207027	Outer membrane protein HopA (omp6)	HP0229	53,2	9615	425	39	
NP_206827	Outer membrane protein HopD (omp2)[a]	HP0025	78,0	223	22	13	
YP_626764	Outer membrane protein HopD (omp2)[a]	HP0025	75,4	207	20	11	
NP_207500	Outer membrane protein HopE (omp15)	HP0706	30,2	425	36	34	x
NP_222958	Outer membrane protein HopF (omp7)	HP0252	52,7	500	33	19	x
NP_207052	Outer membrane protein HopG (omp8)	HP0254	47,5	1289	101	27	x
NP_207947	Outer membrane protein HopI (omp25)	HP1156	76,7	1319	55	31	x
YP_627195	Outer membrane protein HopJ (omp12)	HP0477	41,6	266	13	6	
YP_627837	Outer membrane protein HopL (omp26)	HP1157	133,6	559	42	9	
NP_207025	Outer membrane protein HopM (omp5)	HP0227	76,0	2820	119	18	x
NP_207968	Outer membrane protein HopQ (omp27)[a]	HP1177	70,1	1934	134	13	x
AAY53619	Outer membrane protein HopQ (omp27)[a]	HP1177	68,4	3862	284	21	x
NP_222838	Outer membrane protein HorB (omp4)	HP0127	32,0	630	49	26	x
NP_223078	Outer membrane protein HorD	HP1066	23,5	193	20	28	
NP_207270	Outer membrane protein HorE (omp11)	HP0472	21,0	4776	459	52	x
NP_223332	Outer membrane protein HorF (omp14)	HP0671	30,5	1865	118	46	x
NP_207589	Outer membrane protein HorG (omp18)	HP0796	33,4	92	3	7	
NP_208260	Outer membrane protein HorJ (omp31)	HP1469	28,1	6707	329	41	x
ABQ45555	Outer membrane protein HorK (omp32)	HP1501	36,5	2924	289	58	x
NP_208186	Outer membrane protein HorL (omp30)	HP1395	26,7	913	268	33	x
**Hof family of outer membrane proteins**							
YP_627105	Outer membrane protein HofB	HP1083	53,5	326	16	20	
YP_627203	Outer membrane protein HofC	HP0486	59,8	6196	444	45	x
NP_207284	Outer membrane protein HofD	HP0487	55,4	209	29	22	x
NP_207575	Outer membrane protein HofE	HP0782	52,0	117	11	20	
NP_207581	Outer membrane protein HofF	HP0788	57,0	549	34	35	x
YP_627638	Outer membrane protein HofG	HP0914	58,8	969	56	46	x
NP_223811	Outer membrane protein HofH	HP1167	52,8	236	14	26	x
**Hom family of outer membrane proteins**							
ABV44139	Outer membrane protein HomA	HP0710	72,9	4922	322	31	x
ABV44069	Outer membrane protein HomB	jhp0870[b]	73,5	4602	332	30	x
NP_207171	Outer membrane protein HomC	HP0373	78,7	1343	84	22	
YP_628120	Outer membrane protein HomD	HP1453	82,9	2425	189	28	x
**Iron-regulated outer membrane proteins**							
NP_223344	Iron (III) dicitrate transport protein FecA-1	HP0686	87,8	59	3	5	
NP_208191	Iron (III) dicitrate transport protein FecA-3	HP1400	95,3	1834	122	32	x
NP_223528	Iron-regulated outer membrane protein FrpB1	HP0876	89,2	1650	110	28	x
YP_627639	Iron-regulated outer membrane protein FrpB2	HP0915	91,3	919	63	25	x
NP_208303	Iron-regulated outer membrane protein FrpB3	HP1512	97,5	12816	731	43	x
**Efflux pump outer membrane proteins and other outer membrane proteins**							
NP_207400	Outer-membrane protein of the HefABC efflux system	HP0605	54,6	4735	225	57	x
NP_207762	Outer-membrane protein of the HefDEF efflux system	HP0971	47,7	627	49	51	
NP_207303	Putative outer membrane protein	HP0506	45,9	120	7	19	
NP_223318	Protective surface antigen D15	HP0655	101,7	1866	137	26	x
YP_627420	Lipid A 3'-O-deacylase	HP0694	38,6	1761	74	41	
YP_627451	Predicted outer membrane protein	HP0726	34,3	140	10	12	
NP_208258	Predicted outer membrane protein	HP1467	26,2	272	17	34	
YP_627565	Outer membrane protein P1	HP0839	63,8	1261	70	36	x
NP_207916	Peptidoglycan associated lipoprotein precursor (omp18)	HP1125	20,2	570	39	50	x
NP_224067	Lipoprotein Lpp20	HP1456	19,2	25727	1819	70	x

Gene bank accession number	Protein name	Gene no.	Mass (kDa)	Mowse score	No. of peptides	Sequence coverage (%)	MSII
**CELLULAR PROCESSES**							
**Cell wall/membrane biogenesis**							
YP_628038	Carboxyl-terminal protease	HP1350	50,8	1101	94	44	x
NP_223685	Cyclopropane fatty acid synthase	HP0416	45,8	90	10	18	
NP_207532	D-alanyl-alanine synthetase A	HP0738	40,0	152	8	17	
NP_206887	Hypothetical protein	HP0087	52,4	395	22	35	x
NP_207401	Membrane fusion protein of the HefABC efflux system	HP0606	25,9	1105	43	64	
NP_207761	Nickel-cobalt-cadmium resistance protein NccB	HP0970	39,7	100	7	17	
NP_207578	Outer-membrane lipoprotein carrier protein LolA	HP0785	21,5	102	17	22	
NP_207296	Phospholipase A1 precursor	HP0499	42,7	407	32	14	
ABU80582	Predicted integral membrane protein	HP0983	30,5	222	12	26	
NP_208362	Rare lipoprotein A	HP1571	35,6	794	55	48	
NP_208363	Regulatory protein DniR	HP1572	43,1	56	2	6	
NP_208133	Siderophore-mediated iron transport protein TonB	HP1341	31,6	175	10	32	
YP_627371	Soluble lytic murein transglycosylase	HP0645	65,5	468	29	26	
YP_626931	UDP-3-O-glucosamine N-acyltransferase	HP0196	36,8	590	42	10	
NP_223311	UDP-N-acetylglucosamine 1-carboxyvinyltransferase	HP0648	46,2	56	5	12	
NP_208280	TolC like protein[c]	HP1489	57,0	397	30	34	x
**Cell motility**							
NP_223561	Flagellar biosynthesis protein FlgD	HP0907	40,9	1452	84	53	
AAC35999	Flagellar capping protein FliD	HP0752	74,0	3526	201	56	x
AAA92803	Flagellar hook protein FlgE	HP0870	76,3	2659	115	34	x
YP_627631	Flagellar hook protein FlgE_2	HP0908	67,0	513	39	36	
YP_627798	Flagellar hook-associated protein FlgK	HP1119	68,4	498	40	34	
NP_207093	Flagellar hook-associated protein FlgL	HP0295	91,9	1139	75	53	x
NP_222952	Flagellar P-ring protein precursor FlgI	HP0246	36,6	149	8	19	
NP_223266	Flagellin FlaA	HP0601	53,3	6961	216	46	
NP_222828	Flagellin FlaB	HP0115	53,9	1097	75	41	x
NP_222995	Putative vacuolating cytotoxin (VacA) paralog	HP0289	313,7	133	8	2	
YP_627331	Putative vacuolating cytotoxin (VacA)-like protein	HP0610	348,5	1173	83	11	x
NP_208268	Flagellar basal body P-ring biosynthesis protein FlgA[d]	HP1477	24,4	232	14	31	
YP_626844	Chemotaxis receptor TlpB[e]	HP0103	62,8	373	33	26	
AAB47275	Chemotaxis receptor TlpB[e]	HP0599	48,7	292	22	26	
NP_222812	Methyl-accepting chemotaxis protein TlpA[e]	HP0099	75,1	594	42	29	
YP_626824	Receptor protein of chemotaxis signaliing system TlpC[e]	HP0082	75,3	463	29	24	
**Cell cycle control, mitosis, meiosis**							
NP_207129	Cell division inhibitor	HP0331	29,5	359	14	33	
NP_207769	Cell division protein	HP0978	54,7	78	7	12	
AAC04558	Rod shape-determining protein MreB	HP1373	37,5	454	29	38	
**Defence mechanisms**							
NP_208279	Conserved hypothetical secreted protein	HP1488	36,2	339	17	41	
AAC14433	RND pump protein of the hefABC efflux system	HP0607	113,3	179	13	8	
**Signal transduction mechanisms**							
NP_223705	Chemotaxis protein CheV3	HP0393	35,8	75	3	10	
NP_207278	GTP-binding protein, fusA-homolog	HP0480	66,8	170	29	29	
**Intracellular trafficking and secretion**							
CAA10656	DNA transformation competence protein ComB9	HP0039	37,7	520	27	29	
NP_224168	Protein export protein SecF	HP1549	36,2	154	11	21	
NP_208241	Putative IM protein translocase component YidC	HP1450	62,6	59	8	12	
NP_207371	Signal peptidase I	HP0576	33,8	190	11	27	
NP_207943	Signal recognition particle protein Ffh	HP1152	49,3	429	26	33	
NP_207556	Signal recognition particle receptor FtsY	HP0763	32,5	511	28	49	x

Gene bank accession number	Protein name	Gene no.	Mass (kDa)	Mowse score	No. of peptides	Sequence coverage (%)	MSII
**Posttranslational modification, protein turnover, chaperones**							
YP_628253	Alkyl hydroperoxide reductase	HP1563	22,4	454	42	40	x
YP_627007	ATP-dependent protease binding subunit/heat shock prot	HP0264	96,4	553	36	15	
YP_628066	ATP-dependent protease Lon	HP1379	94,3	73	10	10	
AAC24211	Bifunctional methionine sulfoxide reductase A/B protein	HP0224	41,6	1156	154	42	x
YP_627755	Cytochrome c-type biogenesis protein	HP0378	106,5	104	4	5	
NP_222937	Disulphide isomerase	HP0231	29,6	2314	159	54	x
NP_222731	Heat shock protein 10 GroES	HP0011	13,4	77	8	25	
CAA52062	Heat shock protein 60 GroEL	HP0010	58,3	13704	1195	78	x
NP_222917	Heat shock protein 90 HtpG	HP0210	71,3	132	12	20	
NP_222823	Heat shock protein and co-chaperone 24	HP0110	22,3	60	6	23	
NP_222954	Hypothetical protein	HP0248	40,0	293	14	29	
YP_626850	Molecular chaperone and heat shock protein 70 DnaK	HP0109	67,2	791	54	28	
NP_208232	Peptidyl-prolyl cis-trans isomerase B	HP1441	17,8	139	4	20	
NP_206974	Peptidyl-prolyl cis-trans isomerase C	HP0175	34,0	2518	431	66	x
NP_223721	Predicted DsbC-like protein	HP0377	25,8	111	5	13	x
NP_223124	Protease DO (htrA)	HP1019	51,7	4638	362	57	x
NP_223119	Putative co-chaperone with DnaK	HP1024	32,8	129	5	19	
NP_208253	Putative secreted protein involved in flagellar motility	HP1462	20,6	600	39	47	
NP_207618	Thioredoxin reductase	HP0825	34,2	101	5	12	
YP_627521	Trigger factor Tig	HP0795	51,8	719	89	46	x
NP_206869	Urease accessory protein UreF	HP0069	28,7	148	11	24	x
AAA25026	Urease accessory protein UreH	HP0067	30,0	197	26	25	
NP_207180	Zinc-metalloprotease (YJR117W)	HP0382	46,4	245	13	20	
**METABOLISM**							
**Energy production and conversion**							
NP_207386	2-oxoglutarate-acceptor oxidoreductase subunit OorC	HP0591	20,3	156	10	42	
YP_627726	Biotin sulfoxide reductase	HP0407	90,7	256	38	29	x
NP_206938	Conserved hypothetical iron-sulfur protein	HP0138	55,0	373	36	28	
YP_626886	Cytochrome c oxidase diheme subunit	HP0147	32,9	282	19	29	
NP_206857	D-1-pyrroline-5-carboxylate dehydrogenase	HP0056	135,9	203	15	9	
NP_223779	F0F1 ATP synthase subunit alpha	HP1134	55,4	577	39	34	x
NP_223777	F0F1 ATP synthase subunit beta	HP1132	51,5	248	19	34	x
NP_223780	F0F1 ATP synthase subunit delta	HP1135	20,4	124	6	21	
NP_207924	F0F1 ATP synthase subunit gamma	HP1133	34,2	534	35	41	
NP_207902	Ferrodoxin oxidoreductase beta subunit	HP1111	35,5	272	11	29	
NP_208117	Fumarate hydratase	HP1325	51,4	284	20	27	x
NP_206992	Fumarate reductase, cytochrome b subunit	HP0193	28,9	348	13	17	
YP_626927	Fumarate reductase, flavoprotein subunit	HP0192	80,9	424	84	30	x
NP_206990	Fumarate reductase, iron-sulfur subunit	HP0191	28,5	424	84	25	
NP_206917	Hypothetical protein	HP0117	34,9	60	3	6	
NP_206829	Isocitrate dehydrogenase	HP0027	47,8	210	22	32	x
YP_627947	NADH dehydrogenase subunit C	HP1262	31,7	171	14	27	
NP_208060	NADH dehydrogenase subunit I	HP1268	25,4	167	11	30	
NP_207899	Pyruvate flavodoxin oxidoreductase subunit gamma	HP1108	21,0	108	2	12	
NP_207426	Quinone-reactive Ni/Fe hydrogenase, large subunit	HP0632	64,9	884	47	38	x
NP_206828	Type II citrate synthase	HP0026	48,6	274	16	23	
NP_208330	Ubiquinol cytochrome c oxidoreductase, cytochrome b	HP1539	47,8	109	7	5	
BAF36625	Ubiquinol cytochrome c oxidoreductase, cytochrome c1	HP1538	31,9	265	41	30	
NP_222809	2-hydroxyacid dehydrogenase[f]	HP0096	35,0	93	7	21	
**Carbohydrate transport and metabolism**							
NP_207073	ATP-dependent nuclease	HP0275	50,0	243	13	28	x
NP_208176	Fructose-1,6-bisphosphatase	HP1385	33,1	326	20	36	
NP_206975	Fructose-bisphosphate aldolase	HP0176	33,9	182	17	33	
NP_223746	Glucokinase	HP1103	37,3	261	22	24	
NP_208138	Glyceraldehyde-3-phosphate dehydrogenase	HP1346	36,3	118	11	22	
NP_207879	Transketolase A	HP1088	71,2	48	4	5	

Gene bank accession number	Protein name	Gene no.	Mass (kDa)	Mowse score	No. of peptides	Sequence coverage (%)	MSII
**Amino acid transport and metabolism**							
NP_207828	3-dehydroquinate dehydratase AroQ AroD	HP1038	18,5	113	6	20	
NP_208189	Alanine dehydrogenase	HP1398	41,3	71	3	10	
NP_207443	Aspartase AspA	HP0649	52,5	74	3	8	
NP_223333	Aspartate aminotransferase AspB	HP0672	43,1	141	8	14	
NP_223004	Dipeptide transport substrate-binding protein	HP0298	61,8	297	26	27	
AAO72530	Gamma-glutamyltranspeptidase (ggt)	HP1118	61,1	2584	204	33	x
NP_223179	Glutamine synthetase GlnA	HP0512	54,8	745	32	35	x
YP_627290	Leucyl aminopeptidase PepA	HP0570	55,1	762	39	40	x
YP_627187	Oligoendopeptidase F	HP0470	68,2	160	56	23	
NP_207827	Predicted proline peptidase PepQ	HP1037	41,0	77	6	11	
NP_207420	Tetrahydrodipicolinate N-succinyltransferase	HP0626	44,6	103	10	19	
NP_222790	Urease subunit UreA	HP0073	26,6	4455	404	80	x
AAU21200	Urease subunit UreB	HP0072	61,6	10585	505	59	x
YP_627075	Ketol-acid reductoisomerase IlvC[g]	HP0330	36,7	151	12	24	
NP_207517	L-asparaginase II AnsB[h]	HP0723	35,5	750	52	31	
NP_207099	Dipeptide ABC transporter, ATP-binding protein[i]	HP0301	32,4	53	4	9	
YP_627664	Amino acid ABC transporter, periplasmic binding protein[j]	HP0940	28,7	168	6	17	
YP_627852	Glutamine ABC transporter, periplasmic Gln-binding prot[j]	HP1172	31,3	638	46	54	
**Nucleotide transport and metabolism**							
NP_222817	2',3'-cyclic-nucleotide 2'-phosphodiesterase	HP0104	65,5	263	25	31	x
NP_207147	CTP synthetase	HP0349	61,1	477	22	24	
NP_223486	Inositol-5-monophosphate dehydrogenase	HP0829	51,8	246	14	21	x
NP_207536	Ribose-phosphate pyrophosphokinase[k]	HP0742	35,0	225	9	26	
NP_207065	Chlorohydrolase[l]	HP0267	45,9	59	3	7	
**Coenzyme transport and metabolism**							
NP_207826	7, 8-dihydro-6-hydromethylpterin-pyrophosphokinase	HP1036	19,3	56	4	15	
NP_206962	Delta-aminolevulinic acid dehydratase	HP0163	36,6	77	7	10	
NP_207194	Predicted 3-octaprenyl-4-hydroxybenzoate carboxy-lyase	HP0396	71,4	91	10	10	
NP_224116	Putative riboflavinspecific-deaminase/reductase	HP1505	39,6	48	4	8	
NP_224207	Pyridoxal phosphate biosynthetic protein	HP1582	30,1	1488	142	47	x
NP_208274	Ubiquinone/menaquinone biosynthesis methyltransferase	HP1483	28,2	166	6	14	
NP_207399	Uroporphyrinogen decarboxylase	HP0604	38,7	184	13	22	
AAF04273	Hypothetical protein[l]	HP0654	41,6	172	12	23	x
**Lipid transport and metabolism**							
NP_223287	4-hydroxy-3-methylbut-2-en-1-yl diphosphate synthase	HP0625	39,5	83	5	11	
YP_627416	Acetyl coenzyme A acetyltransferase	HP0690	41,7	113	6	17	
NP_207352	Acetyl-CoA carboxylase subunit alpha	HP0557	35,1	198	11	21	
NP_207742	Acetyl-CoA carboxylase subunit beta	HP0950	32,9	129	8	20	
NP_206890	Acyl-carrier-protein S-malonyltransferase	HP0090	34,9	71	3	7	
NP_207168	Biotin carboxylase	HP0370	51,3	114	7	15	
YP_626930	Enoyl-(acyl carrier protein) reductase	HP0195	30,2	191	10	25	
NP_223026	Predicted membrane bound endonuclease	HP0323	20,4	341	19	51	
NP_206989	Predicted phospholipase D-family protein	HP0190	58,3	208	21	28	x
YP_626936	Putative glycerol-3-phosphate acyltransferase	HP0201	37,0	978	71	47	x
NP_207356	3-ketoacyl-(acyl-carrier-protein) reductase[m]	HP0561	26,7	402	25	42	x
NP_207804	7-alpha-hydroxysteroid dehydrogenase[m]	HP1014	28,6	64	2	9	x

Gene bank accession number	Protein name	Gene no.	Mass (kDa)	Mowse score	No. of peptides	Sequence coverage (%)	MSII
**Inorganic ion transport and metabolism**							
YP_628266	ABC transporter, ATP-binding protein	HP1576	37,0	121	7	22	
NP_223829	Alpha carbonic anhydrase	HP1186	28,4	190	24	36	x
AAA93043	Cadmium-transporting P type ATPase	HP0791	75,7	329	21	16	
YP_627599	Catalase	HP0875	58,7	6462	672	44	
YP_627202	Catalase like protein	HP0485	36,1	636	49	34	x
NP_208252	Cytochrome c551 peroxidase	HP1461	39,2	669	62	37	
NP_208352	Iron (III) ABC transporter, periplasmic iron-binding prot.	HP1561	37,6	2403	160	44	x
NP_224188	Iron (III) ABC transporter, periplasmic iron-binding prot.	HP1562	37,3	1643	122	31	x
NP_223709	Iron-dependent superoxide dismutase SodF SodB	HP0389	24,7	389	19	47	x
NP_223981	Magnesium and cobalt transport protein CorA	HP1344	37,2	92	4	12	
NP_224165	Na+/H+ antiporter NhaA	HP1552	47,9	59	2	5	
NP_207447	Nonheme iron-containing ferritin Pfr	HP0653	19,3	2917	224	52	x
YP_628254	Putative outer membrane lipoprotein PlpA	HP1564	30,3	251	25	41	x
**INFORMATION STORAGE AND PROCESSING**							
**Translation**							
NP_223837	30S ribosomal protein S12	HP1197	15,2	70	2	14	x
NP_208345	30S ribosomal protein S2	HP1554	30,9	245	24	34	
NP_208086	30S ribosomal protein S4	HP1294	24	81	13	33	x
NP_223940	30S ribosomal protein S5	HP1302	16,5	103	4	22	
YP_627930	30S ribosomal protein S6	HP1246	17	56	23	28	x
NP_223836	30S ribosomal protein S7	HP1196	18	103	4	28	
NP_223943	30S ribosomal protein S8	HP1305	15,2	114	3	17	
NP_222798	30S ribosomal protein S9	HP0083	14,5	123	3	27	
NP_207992	50S ribosomal protein L1	HP1201	25,3	219	25	50	
NP_207991	50S ribosomal protein L10	HP1200	18,7	235	33	35	
NP_223842	50S ribosomal protein L11	HP1202	15,4	88	3	22	
NP_206884	50S ribosomal protein L13	HP0084	16,2	265	33	44	x
NP_208093	50S ribosomal protein L15	HP1301	14,7	435	25	32	
NP_208104	50S ribosomal protein L16	HP1312	16,1	253	16	42	x
NP_208108	50S ribosomal protein L2	HP1316	30,3	766	70	53	x
NP_222837	50S ribosomal protein L20	HP0126	14,1	644	21	39	
NP_208287	50S ribosomal protein L25/general stress protein Ctc	HP1496	19,9	302	29		x
NP_208099	50S ribosomal protein L5	HP1307	20,3	1242	115	44	
NP_208096	50S ribosomal protein L6	HP1304	19,5	599	37	39	
YP_627229	50S ribosomal protein L9	HP0514	16,5	473	34	60	
NP_207986	Elongation factor G EF-G	HP1195	77,4	948	46	37	x
NP_208346	Elongation factor Ts EF-Ts	HP1555	39,9	662	47	43	
NP_223846	Elongation factor Tu EF-Tu	HP1205	43,8	785	32	28	
NP_206981	Lysyl-tRNA synthetase	HP0182	57,9	83	2	4	
NP_223937	Methionine aminopeptidase	HP1299	27,8	145	8	19	
NP_207201	Phenylalanyl-tRNA synthetase subunit alpha	HP0403	38,5	113	8	14	
YP_627731	Phenylalanyl-tRNA synthetase subunit beta	HP0402	86,3	140	8	10	x
NP_206923	Threonyl-tRNA synthetase	HP0123	70,6	102	3	5	
NP_207839	Translation initiation factor IF-2	HP1048	105,3	97	9	8	
NP_206924	Translation initiation factor IF-3	HP0124	23,5	65	3	15	
**Transcription**							
YP_627979	DNA-directed RNA polymerase subunit alpha	HP1293	38,6	57	6	12	
BAA84590	RNA polymerase sigma 70	HP0088	78,3	63	3	4	
**Replication, recombination and repair**							
YP_627508	Hypothetical protein	HP0781	49,6	322	13	19	x
NP_206952	Recombinase A	HP0153	37,8	787	33	40	

Gene bank accession number	Protein name	Gene no.	Mass (kDa)	Mowse score	No. of peptides	Sequence coverage (%)	MSII
**POORLY CHARACTERIZED**							
**General function prediction only**							
NP_223000	Aliphatic amidase AmiE	HP0294	38,5	465	39	37	
NP_208169	Competence lipoprotein ComL	HP1378	26,3	62	5	12	
NP_208221	Conserved hypothetical ATP-binding protein	HP1430	77,6	939	63	42	
NP_207908	Conserved hypothetical secreted protein	HP1117	29,3	71	11	22	x
ABQ45658	Cystein rich protein D	HP0160	35	327	22	24	
NP_206886	Hypothetical protein	HP0086	51,2	493	38	31	
ABQ45759	Hypothetical protein	HP0628	39,1	159	18	15	
NP_207539	Hypothetical protein	HP0746	48,1	532	34	38	
NP_223428	Hypothetical protein	HP0773	40,1	169	10	25	x
NP_208248	Hypothetical protein	HP1457	23	12826	386	50	x
NP_207683	Predicted short-chain oxidoreductase	HP0890	29,1	158	7	12	
NP_207451	Processing protease	HP0657	48,8	1557	79	48	x
NP_207802	Protease PqqE	HP1012	50,3	637	64	45	x
NP_207033	Putative b-lactamase precursor HcpE	HP0235	40,7	207	17	16	
**Function unknown**							
YP_627968	Conserved hypothetical secreted protein	HP1286	20,6	2174	162	48	
YP_626759	Hypothetical protein	HP0018	53,7	540	46	37	
NP_222846	Hypothetical protein	HP0137	23,7	80	6	23	
NP_207630	Hypothetical protein	HP0837	11,3	142	22	24	
NP_207915	Hypothetical protein	HP1124	38,4	690	58	48	
NP_208359	Hypothetical protein	HP1568	21,1	471	26	58	
NP_208379	Hypothetical protein	HP1588	28,7	1789	91	55	x
**PROTEINS NOT CLASSIFIED**							
YP_627754	Alpha 1,3-fucosyltransferase FucT	HP0651	54,0	70	4	7	
AAF80191	Cag patogenicity island protein 1 (cag1)	HP0520	12,6	240	8	33	
P80200	Cag patogenicity island protein A (cag26)	HP0547	128,0	2040	240	45	x
NP_207339	Cag patogenicity island protein F (cag22)	HP0543	31,8	73	7	15	
NP_207333	Cag patogenicity island protein M (cag16)	HP0537	43,8	107	15	18	
NP_207328	Cag patogenicity island protein T (cag12)	HP0532	32,5	85	7	30	
Q48261	Neuraminyllactose-binding hemagglutinin precursor	HP0797	29,2	4226	332	69	x
NP_207768	Peptidyl-prolyl cis-trans isomerase D	HP0977	56,5	181	13	22	
CAD79440	Plasminogen binding protein PgbA	HP0508	52,2	663	74	41	x
ABQ08061	Put. neuraminyllactose-binding hemagglutinin homolog	HP0410	28,4	934	107	47	x
YP_627209	Put. neuraminyllactose-binding hemagglutinin homolog	HP0492	31,9	694	79	27	x
YP_627317	Tumor necrosis factor a-inducing protein	HP0596	22,1	1851	94	47	x
Q48247	Vacuolating cytotoxin VacA	HP0887	139,8	2569	173	36	x
**HYPOTHETICAL PROTEINS NOT CLASSIFIED**							
YP_626775	Hypothetical protein	HP0036	38,6	82	4	12	
NP_222796	Hypothetical protein	HP0080	65,8	623	40	25	x
NP_206897	Hypothetical protein	HP0097	26,1	663	65	33	x
YP_626860	Hypothetical protein	HP0120	47,4	96	12	10	
NP_222839	Hypothetical protein	HP0129	16,7	510	58	27	x
NP_206930	Hypothetical protein	HP0130	32,9	1713	280	36	x
NP_206969	Hypothetical protein	HP0170	28,7	138	5	20	
YP_626923	Hypothetical protein	HP0185	30,6	241	8	19	
NP_222938	Hypothetical protein	HP0232	23,0	64	10	26	
NP_207103	Hypothetical protein	HP0305	20,4	5342	255	54	x
NP_207165	Hypothetical protein	HP0367	23,2	130	15	43	
YP_627717	Hypothetical protein	HP0418	39,9	133	8	17	
NP_223136	Hypothetical protein	HP0466	29,9	188	11	18	
YP_627273	Hypothetical protein	HP0554	37,2	110	6	15	
YP_627274	Hypothetical protein	HP0555	31,3	91	8	31	
YP_627283	Hypothetical protein	HP0563	49,3	513	23	26	x

Gene bank accession number	Protein name	Gene no.	Mass (kDa)	Mowse score	No. of peptides	Sequence coverage (%)	MSII
YP_627325	Hypothetical protein	HP0603	23,0	126	13	20	
NP_207453	Hypothetical protein	HP0659	47,6	926	50	31	x
NP_207515	Hypothetical protein	HP0721	17,6	834	60	35	x
YP_627510	Hypothetical protein	HP0783	20,0	156	5	20	
YP_627587	Hypothetical protein	HP0863	63,7	1357	103	35	x
NP_223534	Hypothetical protein	HP0884	71,4	79	4	7	
NP_207745	Hypothetical protein	HP0953	21,4	212	12	34	
NP_223625	Hypothetical protein	HP0973	41,0	53	3	10	
NP_223120	Hypothetical protein	HP1023	46,9	295	9	17	
YP_627133	Hypothetical protein	HP1055	35,0	1208	64	30	x
YP_627132	Hypothetical protein	HP1056	33,1	555	34	27	
NP_223087	Hypothetical protein	HP1057	27,3	308	13	18	x
NP_207964	Hypothetical protein	HP1173	20,8	768	45	43	x
YP_628202	Hypothetical protein	HP1454	34,2	13744	829	53	x
NP_208254	Hypothetical protein	HP1463	25,0	432	29	39	x
NP_208315	Hypothetical protein	HP1525	24,9	466	36	21	
NP_208337	Hypothetical protein	HP1546	19,7	59	2	14	
NP_223662	Hypothetical protein	jhp0945[b]	73,3	52	2	3	

[a]=Protein can be encoded by two alleles, sequence distance below 90%

[b]=No homolog in 26695

[c]=Also classified in COG as: Intracellular trafficking and secretion

[d]=Also classified in COG as: Posttranslational modification, protein turnover, chaperones

[e]=Also classified in COG as: Signal transduction mechanisms

[f]=Also classified in COG as: Coenzyme transport and metabolism//General function prediction only

[g]=Also classified in COG as: Coenzyme transport and metabolism

[h]=Also classified in COG as: INFORMATION STORAGE AND PROCESSING: Translation

[i]=Also classified in COG as: Inorganic ion transport and metabolism

[j]=Also classified in COG as: CELLULAR PROCESSES: Signal transduction mechanisms

[k]=Also classified in COG as: Amino acid transport and metabolism

[l]=Also classified in COG as: General function prediction only

[m]=Also classified in COG as: General function prediction only//Secondary metabolites biosynthesis, transport and catabolism

Table S2: MS II - *H. pylori* vesicle proteins not found in MS I

Gene bank accession number	Protein name	Gene no.	Mass (kDa)	Mowse score	No. of peptides	Sequence coverage (%)
**OUTER MEMBRANE PROTEINS**						
**Major outer membrane protein**						
NP_208134	Outer membrane protein (omp29)	HP1342	76	420	14	12,3
AAD06240	Outer membrane protein (omp16)	HP0722	72,4	214	19	10,4
**CELLULAR PROCESSES**						
**Intracellular trafficking and secretion**						
AAF04275	TolB	HP1126	47,7	344	32	41
**METABOLISM**						
**Energy production and conversion**						
ABF84636	2-oxoglutarate oxidoreductase OorA subunit	HP0589	41,6	50	4	9,9
**Carbohydrate transport and metabolism**						
NP_208286	Transaldolase	HP1495	35,6	46	4	11,1
**INFORMATION STORAGE AND PROCESSING**						
**Translation**						
ABF85330	Ribosomal protein L4 (rplD)	HP1318	24,2	84	10	25,6
**Replication, recombination and repair**						
AAA74376	Gyrase A	HP0701	92,6	89	2	2,8
**POORLY CHARACTERIZED**						
**General function prediction only**						
AAD06784	Putative acid phosphatase lipoprotein	HP1285	27,9	82	6	9,4
NP_207571	Hypothetical protein	HP0778	26,9	56	5	17,6

Fig. S3

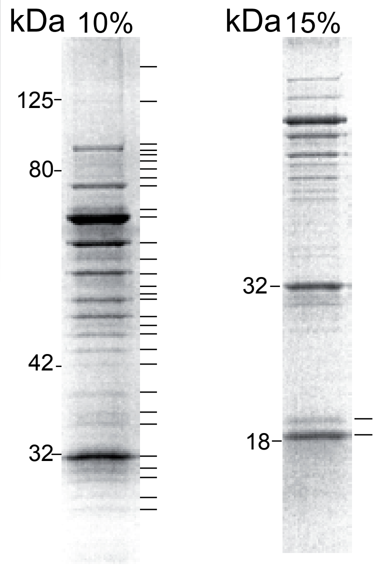

MS II analysis
A fraction of *H. pylori* vesicles corresponding to densities between 1.17 and 1.18 g/ml was separated with 10% and 15% SDS-PAGE, respectively.
A series of 32 bands were excised and subjected to nanoflow LC FT-ICR MS/MS.
The excised bands are marked with bars to the right of the gels.
The same proteins identified in MS II as in MS I are indicated in Table S1 and proteins present only in MS II are presented in Table S2.

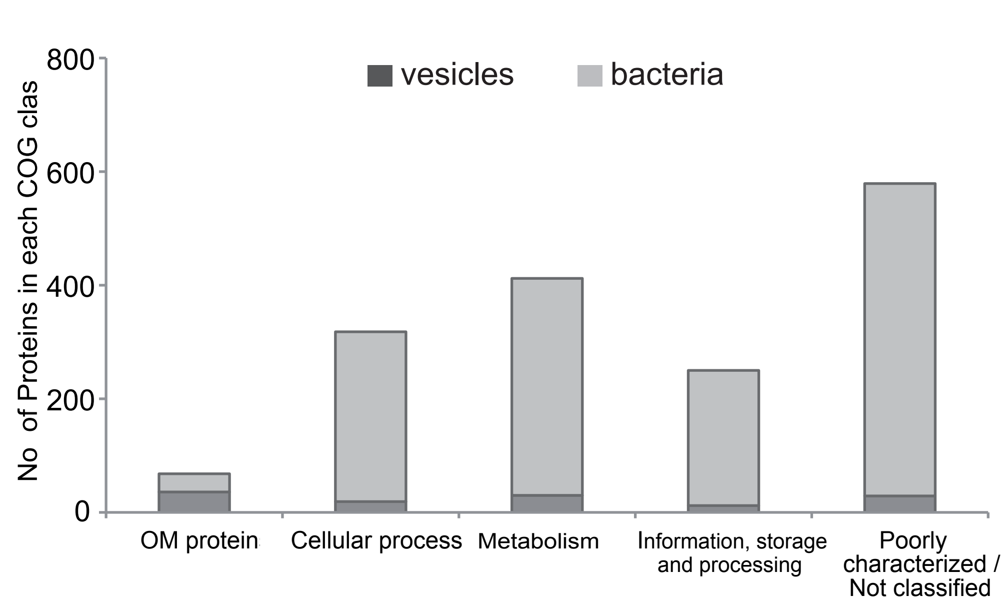

COG classification of proteins identified in MS II
All proteins present in strain 26695 are COG classified into 4 classes, an extra group of OM proteins is added (See experimental procedures).
Dark grey bar represents vesicle proteins in each class and light grey bars represent number of proteins of each class of the total *H. pylori* proteome.
Out of the total *H. pylori* proteome, 53% of all OM proteins were found in the vesicles;
6% of Cellular processes;
7% of Metabolism;
5% of Information, Storage and Processing; and
5% of Poorly classified / Not classified.

Fig. S4

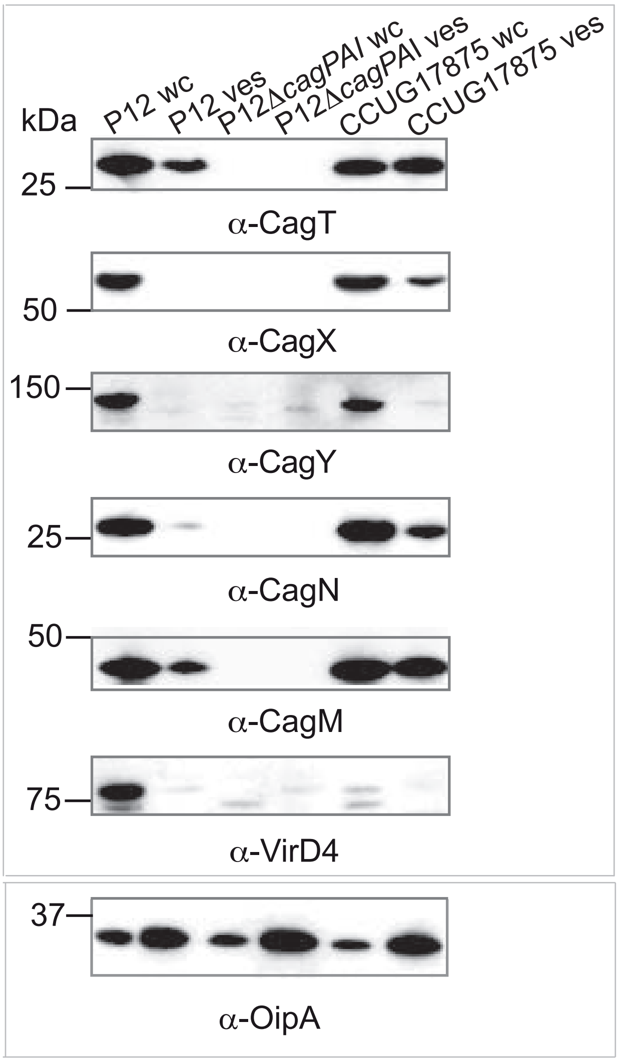

T4S associated proteins in H. pylori vesicles
Protein extracts of *H. pylori* cells (wc) and vesicles (ves) (5μg) from strains P12; P12Δ*cagPAI*; and CCUG17875 were probed for a series of T4S secretion associated proteins using antiserum against CagT, CagX, CagY, CagN, CagM and VirD4. CagT, CagX, CagN and CagM were all present in vesicles from strain CCUG17875. VirD4 is present in whole bacteria from strain P12 but no protein was detected in P12 vesicles.

OipA protein in H. pylori vesicles
Protein extracts of the same series of strains as above probed with anti-OipA antibodies. OipA is present in both bacterial cells and vesicles.

Fig. S5

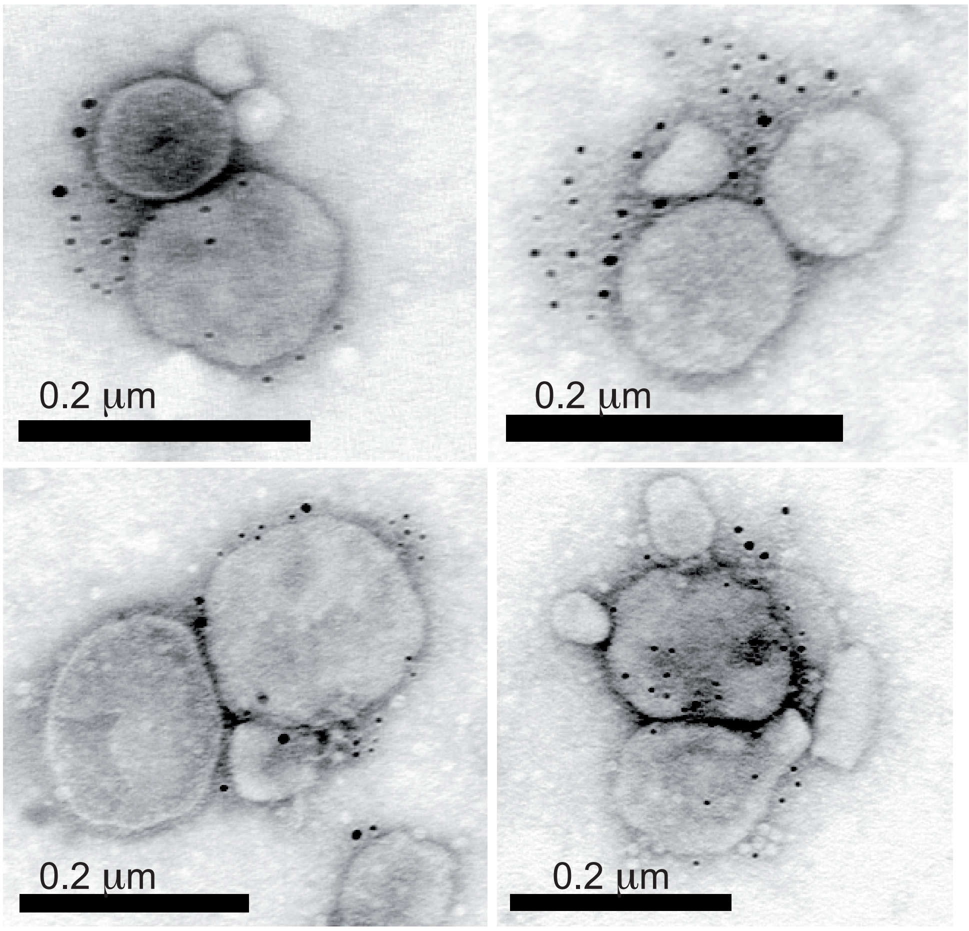

Presence of the BabA and SabA adhesins on the surface of the same H. pylori *vesicle*
Electron micrograpghs of *H. pylori* vesicles. The BabA adhesins are visualized by anti-BabA antibodies and 10 nm gold particles and the SabA adhesins are visualized with sLex receptor conjugate and 5 nm gold particles. Bar length = 0.2 μm

Fig S6

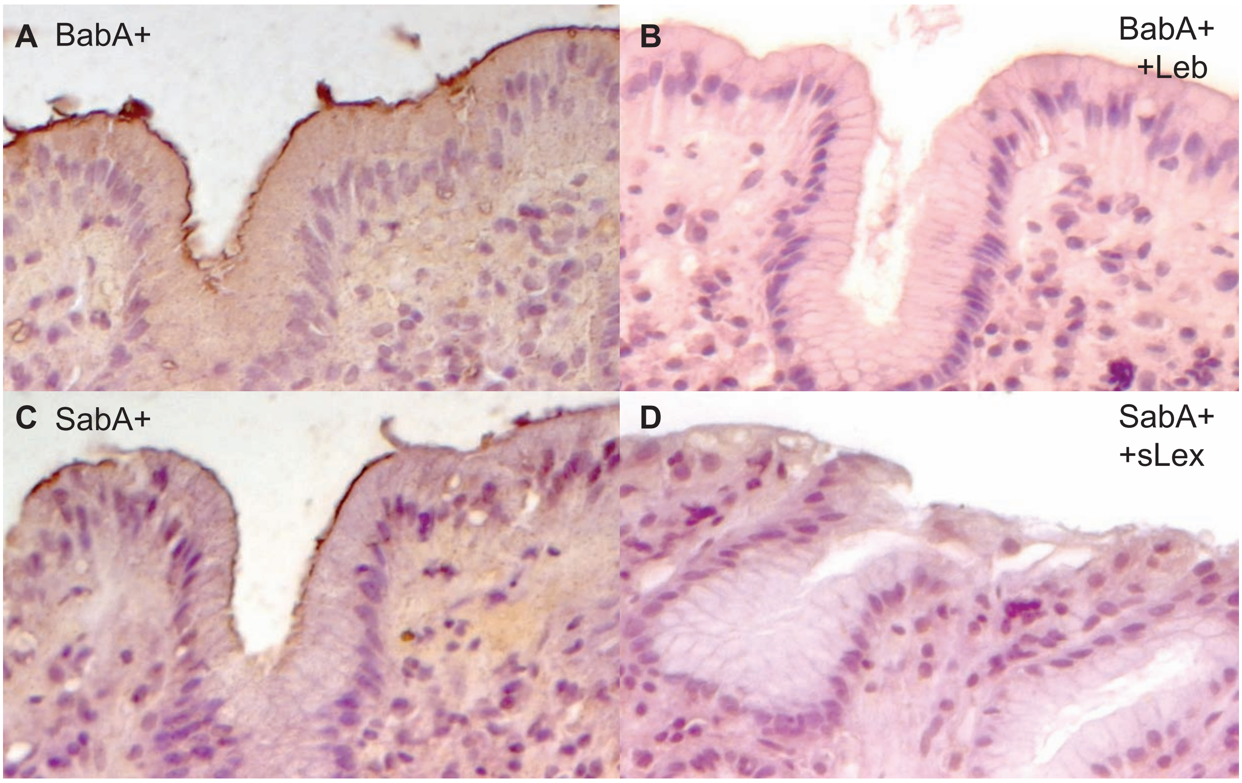

Vesicle adherence to the epithelium of human gastric mucosa via BabA–Leb interactions and SabA-sLex interactions was detected using biotinylated anti-*H. pylori* antibody and streptavidin-ABComplex and DAB. Vesicles isolated from *H. pylori* strains 17875/Leb (BabA+/SabA-) and 17875/sLex (BabA-/SabA+) were incubated with human gastric tissue sections as follows:

A) 17875/Leb vesicles;

B) 17875/Leb vesicles pre-incubated with Leb receptor conjugate prior to incubation with gastric tissue sections;

C) 17875/sLex vesicles;

D) 17875/sLex vesicles pre-incubated with sLex receptor conjugate prior to incubation with gastric tissue sections.

Tissue sections were counterstained with Mayers hematoxylin.
